# The Phenotypic and Genetic Underpinnings of Flower Size in Polemoniaceae

**DOI:** 10.3389/fpls.2015.01144

**Published:** 2016-01-05

**Authors:** Jacob B. Landis, Rebecca D. O'Toole, Kayla L. Ventura, Matthew A. Gitzendanner, David G. Oppenheimer, Douglas E. Soltis, Pamela S. Soltis

**Affiliations:** ^1^Department of Biology, University of FloridaGainesville, FL, USA; ^2^Florida Museum of Natural History, University of FloridaGainesville, FL, USA; ^3^Genetics Institute, University of FloridaGainesville, FL, USA; ^4^Plant Molecular and Cellular Biology Graduate Program, University of FloridaGainesville, FL, USA

**Keywords:** flower epidermal cells, comparative transcriptomics, pollinator-mediated selection, Polemoniaceae, floral evo-devo, phylogeny, *Saltugilia*

## Abstract

Corolla length is a labile flower feature and has strong implications for pollinator success. However, the phenotypic and genetic bases of corolla elongation are not well known, largely due to a lack of good candidate genes for potential genetic exploration and functional work. We investigate both the cellular phenotypic differences in corolla length, as well as the genetic control of this trait, in *Saltugilia* (Polemoniaceae). Taxa in this clade exhibit a large range of flower sizes and differ dramatically in pollinator guilds. Flowers of each species were collected from multiple individuals during four stages of flower development to ascertain if cell number or cell size is more important in determining flower size. In *Saltugilia*, increased flower size during development appears to be driven more by cell size than cell number. Differences in flower size between species are governed by both cell size and cell number, with the large-flowered *S. splendens* subsp. *grantii* having nearly twice as many cells as the small-flowered species. Fully mature flowers of all taxa contain jigsaw cells similar to cells seen in sepals and leaves; however, these cells are not typically found in the developing flowers of most species. The proportion of this cell type in mature flowers appears to have substantial implications, comprising 17–68% of the overall flower size. To identify candidate genes responsible for differences in cell area and cell type, transcriptomes were generated for two individuals of the species with the smallest (*S. australis*) and largest (*S. splendens* subsp. *grantii*) flowers across the same four developmental stages visualized with confocal microscopy. Analyses identified genes associated with cell wall formation that are up-regulated in the mature flower stage compared to mid-stage flowers (75% of mature size). This developmental change is associated with the origin of jigsaw cells in the corolla tube of mature flowers. Further comparisons between mature flowers in the two species revealed 354 transcripts that are up-regulated in the large-flowered *S. splendens* subsp. *grantii* compared to the small-flowered *S. australis*. These results are likely broadly applicable to Polemoniaceae, a clade of nearly 400 species, with extensive variation in floral form and shape.

## Introduction

The vast morphological diversity observed in angiosperms is often considered to result in part from the interaction between flowers and their pollinators (e.g., Crepet, 1995; Waser et al., 1996; Crepet and Niklas, 2009; Van der Niet and Johnson, 2012; Kearns et al., 2015). Differences in morphological phenotypes are often associated with different pollinator classes to form pollination syndromes (Fenster et al., 2004) that may comprise many floral traits (Waser, 2006), with many studies showing the selection pressures certain pollinators place on these floral traits (Bruneau, 1997; Johnson et al., 1998; Fulton and Hodges, 1999; Schemske and Bradshaw, 1999; Whittall and Hodges, 2007; Cronk and Ojeda, 2008; Brunet, 2009).

Changes in floral traits associated with different pollination syndromes, especially traits involving size and number of organs, have been observed to be highly labile (Stebbins, 1974; Givnish, 2002; Lock et al., 2011; Alapetite et al., 2014) and are mostly attributed to shifts in timing, rates and/or patterns of gene expression (Pina et al., 2014). One important and labile floral trait associated with pollinator selection, corolla length, has been shown experimentally to be heritable and selectable (Mitchell and Shaw, 1993; Kaczorowski et al., 2008; Gómez et al., 2009). The length of the corolla is often more stable than the spread (width) of the corolla, with the spread being more highly modified under both internal and external stressors (Goodspeed and Clausen, 1915).

Corolla development is typically marked by two phases of growth: early growth involving cell division, followed by cell expansion in later growth (Irish, 2008; Hepworth and Lenhard, 2014). Where organ growth (i.e., corolla size) due to cell expansion is involved, enlargement of the cell wall must occur with either expansion of vacuoles or endoreduplication (Weiss et al., 2005; Anastasiou and Lenhard, 2007). However, the relative contribution of cell division and cell elongation to corolla growth is debated and likely varies among species (Martin and Gerats, 1993; Stuurman et al., 2004), with the shape of petal epidermal cells having been investigated in many angiosperm families (Kay et al., 1981; Ojeda et al., 2009, 2012). Recent experiments show that formation and extension of petal nectar spurs in both *Aquilegia* (Ranunculaceae) and *Centranthus* (Caprifoliaceae) are controlled primarily by cell elongation (Puzey et al., 2012; Mack and Davis, 2015), raising the hypothesis that corolla length may operate under the same genetic control mechanisms. However, the genetic components of flower size, specifically the control of cell number, cell size and cell shape, are not well understood (Glover, 2007; Ojeda et al., 2012).

Indirect evidence primarily from *Arabidopsis thaliana* and *Antirrhinum majus*, representing the evolutionarily divergent lineages of rosids and asterids, respectively, indicates several potential candidate genes that may be involved in corolla elongation, such as *JAGGED, AINTEGUMENTA, ARGOS, BPFP, OPR3, ARF8, BIG BROTHER, KLH, DAI* and *GAST1* (Herzog et al., 1995; Elliott et al., 1996; Kotilainen et al., 1999; Mizukami and Fischer, 2000; Mizukami, 2001; Kim et al., 2002; Ben-Nissan et al., 2004; Disch et al., 2006; Krizek, 2009; Xu and Li, 2011; Chang et al., 2014). Additional studies in *Petunia* have identified at least five QTL involved with corolla tube morphology and elongation (Stuurman et al., 2004; Galliot et al., 2006). Despite the identification of these candidate genes, further studies of expression and function involving any of these genes are lacking in non-model species. To elucidate the phenotypic, developmental and genetic components of flower size in the context of pollination biology, we are focusing on the phlox family, Polemoniaceae.

Polemoniaceae (26 genera, 387 species; Johnson et al., 1996, 2008; Prather et al., 2000; Ferguson and Jansen, 2002; Landis et al., in review) comprise annual and perennial plants native to North and South America, with the center of diversity in western North America (Grant, 1959). This family has a wide diversity of corolla length, varying from 3 mm in the mid-elevation selfing species *Lathrocasis tenerrima* to over 70 mm in species of *Cantua* and *Cobaea* (Schönenberger, 2009; Landis et al., in review). Polemoniaceae have long been a model for studies of pollination biology (e.g., Grant and Grant, 1965). More recent studies have addressed variation in corolla length, with bees selecting for longer tubes and funnel-shaped flowers in *Polemonium* (Galen and Cuba, 2001), increased moisture availability resulting in increased flower size in *Leptosiphon* (Lambrecht, 2013) and longer-flowered individuals excluding bees in four species of *Phlox* (Strakosh and Ferguson, 2005).

The focus of the current study is *Saltugilia*, which comprises four species—*S. australis, S. caruifolia, S. latimeri*, and *S. splendens* (Porter and Johnson, 2000; Johnson, 2007)—that differ primarily in corolla morphology (Weese and Johnson, 2005) and exhibit a 2.5-fold range in corolla size. These taxa also vary in observed pollinators, with *S. australis* and *S. latimeri* being autogamous, *S. caruifolia* pollinated by bees and the different subspecies of *S. splendens* pollinated by hummingbirds (*S. splendens* subsp. *grantii*) and bee flies (*S. splendens* subsp. *splendens*) (Grant and Grant, 1965; Weese and Johnson, 2005).

Our overall goal is to integrate phylogenetic, phenotypic and developmental analysis of floral traits associated with pollination syndromes and observed differences in corolla length, while linking the underlying genetic components of these differences to elucidate the mechanisms of pollinator-mediated selection in representatives of Polemoniaceae. Specifically, we investigated (1) the relationships of all currently recognized taxa in *Saltugilia* by reconstructing the phylogeny with both nuclear and plastid markers to provide a framework for the remaining goals; (2) the cellular component of differences in flower size to address the question of whether differences in flower size are predominantly controlled by cell size or cell number, or a combination of the two; and (3) the genetic underpinnings associated with differences in flower size in *Saltugilia*.

## Materials and methods

### Plant material

Four taxa of *Saltugilia* and one of *Gilia* were grown in the greenhouses at the University of Florida from seeds obtained from Rancho Santa Ana Botanic Garden (Claremont, CA, USA), the Ornamental Germplasm Center at The Ohio State University (Columbus, OH, USA) and Leigh Johnson (Brigham Young University; Provo, UT, USA): *G. brecciarum* subsp. *brecciarum* (W6 30785), *S. australis* (Johnson BYU), *S. caruifolia* (RSABG 19148), *S. splendens* subsp. *grantii* (RSABG 21757) and *S. splendens* subsp. *splendens* (RSABG 22676) (Table 1). Two additional samples of *Saltugilia* and one additional species of *Gilia* were collected in California in the spring of 2015: *G. stellata* (JOTR34200), *S. latimeri* (UCR261592) and a field accession of *S. splendens* subsp. *splendens* (JOTR32513), which differed morphologically from the accession obtained from Rancho Santa Ana Botanic Garden. Collections of *S. splendens* subsp. *splendens* and *G. stellata* were obtained with the help of Tasha LaDoux (University of California Riverside, Riverside, CA, USA), with the above accession numbers for those species representing previous collections from the same localities sampled here.

**Table 1:** **Plant material information, including herbarium accessions, locality of collection for field samples and source of seed material as well as sequencing coverage for phylogenetic analyses**.

**Species**	**Growth facility/Locality collected**	**Accession/Voucher**	**Plastid coding genes/80 total (bp)**	**Plastome length (bp)/percentage of plastome covered (coverage) and coverage**	**Nuclear genes/90 captured (bp)**
*Gilia brecciarum* subsp. *brecciarum*	UF Greenhouse	OGPC W6 30785	80/80 (64,439)	152,899/99.4% (403x)	90/90 (143,769)
*Gilia stellata*	UF Greenhouse	JOTR34200	80/80 (64,495)	153,396/99.7% (926x)	90/90 (141,693)
*Saltugilia australis*	UF Greenhouse	L. Johnson Brigham Young University	73/80 (63,380)	124,673/81.0% (11.4x)	82/90 (90,130)
*Saltugilia caruifolia*	UF Greenhouse	RSABG 19148	62/80 (58,418)	96,715/62.9%(1.7x)	46/90 (34,942)
*Saltugilia latimeri*	Joshua Tree National Park/Big Horn Mountains	UCR261592	80/80 (64,491)	153,396/99.7% (825x)	90/90 (142,909)
*Saltugilia splendens* subsp. *grantii*	UF Greenhouse	RSABG 21757	74/80 (64,178)	132,303/86.0% (5.3x)	60/90 (64,429)
*Saltugilia splendens* subsp. *splendens*	UF Greenhouse	RSABG 22676	79/80 (64,178)	135,926/88.3% (11.2x)	87/90 (106,862)
*Saltugilia splendens* subsp. *splendens*	Joshua Tree National Park	JOTR32513	80/80 (64,497)	153,504/99.8% (1,379x)	90/90 (144,653)

### Phylogenetic analyses

DNA was extracted from samples of all eight taxa following a modified CTAB extraction protocol (Doyle and Doyle, 1987). Rehydrated DNA was sonicated to a targeted length of 300-bp fragments using a Covaris S220 sonicator (Covaris, Inc., Woburn, MA, USA) following the manufacturer's suggested protocol. A total of 3–5 μg of sheared DNA from each sample was sent to RapidGenomics (Gainesville, FL, USA) for Illumina library preparation with dual indexed barcodes and targeted exon capture using MYbaits probes (MYcroarray; Ann Arbor, MI, USA). This approach was used to generate 100 single-copy nuclear genes for phylogenetic analysis. Probes were designed from transcriptomes of four species [*Fouqueria macdouglaii* (Fouquieriaceae)*, Phlox drummondii* (Polemoniaceae)*, Phlox* sp., and *Ternstroemia gymnathera* (Pentaphylacaceae)] available in the 1KP data set (www.onekp.com) and the *Arabidopsis* genome using MarkerMiner (Chamala et al., 2015) to find putative single-copy genes. One hundred nuclear genes were selected with exons of at least 300 bp and introns smaller than 600 bp. Probes were designed for 120 kmers and tiled 3x for additional capture efficiency. Capture products were pooled with additional samples and distributed across two runs: Illumina NextSeq 500 (2 × 150 bp) mid-throughput and an Illumina HiSeq 2000 (2 × 100 bp).

Raw reads were processed using two custom scripts (available on Github; https://github.com/soltislab/get_BLAT_reads). With this pipeline, reads were trimmed and filtered using cutadapt (Martin, 2011) and Sickle (Joshi and Fass, 2011), followed by a BLAT (Kent, 2002) analysis to isolate (1) plastid reads using the complete plastome reference of *Phlox amabilis* (provided by J. Mark Porter, unpublished) and (2) nuclear reads using a representative of each of the 100 nuclear genes. On-target reads were imported to Geneious (version 8.0.5; Biomatters Limited, Auckland, New Zealand) and mapped to the reference sequences (*P. amabilis* plastome for plastid coding genes and the complete plastome; one representative for each of the 100 loci for nuclear genes) (Supplemental Data 1) using the Geneious mapper with the following settings: medium sensitivity with 10 iterations, majority threshold to reduce ambiguities, with no coverage and coverage of less than 2 sequences coded as missing data.

Concatenated consensus reads for each taxon, consisting of 80 coding regions of the plastome, the complete plastome including coding genes and spacer regions and 90 nuclear loci for which there was sufficient coverage and overlap among taxas, were aligned using MAFFT (version 7.245; Katoh and Standley, 2013) installed on the University of Florida Research Computing cluster. Individual alignments of plastid coding genes and nuclear genes were analyzed in PartitionFinder (version 1.1.1; Lanfear et al., 2012) to find the best partitions for maximum likelihood (ML) inference. Separate phylogenetic analyses of the two plastid data sets and nuclear genes were conducted using RAxML (version 8.2.2; Stamatakis, 2014) installed on the University of Florida Research Computing cluster. The two species of *Gilia* were used as outgroups.

Plastid coding regions and nuclear loci were analyzed with 1000 bootstrap replicates with the preferred partitions identified by PartitionFinder for all gene partitions, while the complete plastome was analyzed with 1000 bootstrap replicates using the GTR+G substitution model chosen by jModeltest (version 2.1.1; Darriba et al., 2012). The two separate analyses of the plastome data were conducted to assess the potential impact that missing data had on the phylogenetic reconstructions. Raw reads for each accession were deposited in GenBank's Short Read Archive (http://www.ncbi.nlm.nih.gov/sra) and assembled plastomes and nuclear genes were submitted to GenBank (all accession numbers are given in Supplemental Table 1). Concatenated alignment files for each dataset are deposited in Figshare (DOI: 10.6084/m9.figshare.2007546, 10.6084/m9.figshare.2007549, and 10.6084/m9.figshare.2007555).

Following the completion of phylogenetic analyses, ancestral-state reconstructions of pollinators and flower size were conducted on the resulting topologies. Both a maximum parsimony (MP) and ML framework were implemented using Mesquite (version 3.03; Maddison and Maddison, 2015) for reconstructing pollinators. Pollinator states included autogamous (*G. brecciarum* subsp. *brecciarum, G. stellata, S. australis* and *S. latimeri*), bee pollination (*S. caruifolia*), hummingbird pollination (*S. splendens* subsp. *grantii*) and bee fly pollination (*S. splendens* subsp. *splendens*). For flower size, a continuous reconstruction was conducted in Mesquite using the average flower size of measured flowers, with flower length ranging from 0.8 to 2.5 cm.

### Cell morphology

Flower material of four developmental stages was collected from four individual plants per species when available (all stages and individuals could not be collected from all field samples due to insufficient flowering material on the plant): mature (flowers at anthesis), mid (75% of total flower length at anthesis), half (50% of total flower length at anthesis) and small (25% of total flower length at anthesis). Sample preparation and confocal microscopy were carried out following Landis et al. (2015). Imaging of cells was conducted using the methods of Landis et al. (2015), using a Zeiss Axiocam HRm camera mounted on a Zeiss Axioplan 2 Imaging microscope (Jena, Germany) and Axiovision software. Green fluorescence was obtained using Zeiss filter set 10 (excitation wavelengths, 45–490 nm; dichroic, 510 nm LP; emission wavelengths, 515–565 nm), with a 40x magnification lens and Apotome with optical sectioning with optical slice distance of 0.675 μm. Confocal images were imported into Fiji (http://fiji.sc/Fiji; Schindelin et al., 2012) and up to 50 cells per image were outlined and measured for area, perimeter, shape descriptor (circularity value; Glasbey and Horgan, 1995) and bounding box length and width (the smallest box possible to encompass each cell).

Circularity values were calculated with the following formula: circularity = 4π(area/perimeter^2^). A value of 1.0 indicates a perfect circle, with values approaching 0 indicating increasingly elongated cells. One-way ANOVA and Tukey's honest significance tests were performed in R (version 3.2.1) with generation of box plots for the four developmental stages across all taxa, with data from all individuals per taxon pooled. Pooled data were also used to calculate mean and standard deviation of cell size for each cell type in each stage of development. Estimates of cell number were conducted for each cell type by taking the mean values of the width of the bounding box. The total length of each cell type was then divided by these values to obtain estimates of cell number.

### Transcriptome analysis

Total RNA was extracted from flowers at each of the four developmental stages of two individuals each of the small-flowered *S. australis* and the large-flowered *S. splendens* subsp. *grantii* using Tri-Reagent following the manufacturer's directions (Ambion, Austin, TX, USA). Library preparation of these 16 samples was conducted using the NEBNext Ultra RNA Library Prep Kit for Illumina (NEB, Ipswich, MA, USA) following the manufacturer's directions and NEBNext Multiplex Oligos for Illumina Index Primers Set 1 barcodes (NEB, Ipswich, MA, USA). Transcriptome sequencing was performed at the University of Florida Interdisciplinary Center for Biotechnology Research using two runs on the Illumina NextSeq 500 (2 × 150 bp) mid-throughput with 8 samples pooled per run. Raw reads were trimmed and filtered using the same pipeline described above minus the BLAT analysis. Following cleaning, *de novo* assemblies using the published Trinity pipeline were conducted (Grabherr et al., 2013; Haas et al., 2013).

A reference assembly was generated for each species with all reads from all stages and both individuals pooled. The *S. australis* reference was constructed with 66,850,418 paired-end reads and an additional 3,610,028 singleton reads, while the *S. splendens* subsp. *grantii* reference was constructed with 90,875,540 paired-end reads and 3,922,978 singleton reads. A final reference was created with all raw reads from both species for downstream analysis of gene expression between species. A BLAST analysis was conducted on each reference, *S. australis, S. splendens* subsp. *grantii* and the combined reference, to determine the number of known proteins that match a transcript by at least 80% coverage, with only the top transcript hit for each protein returned (UniProt Consortium, 2015). All raw reads for each species were then mapped back to the reference assemblies using RNA-Seq by Expectation-Maximization as implemented in Trinity, and filtered by 1 fpkm to remove artifacts that were formed in the *de novo* assemblies and to reduce the false discovery rate. Each filtered reference assembly was then used to map raw reads of each developmental stage for each of the two individuals for the respective species. These newly created fasta files were translated into open reading frames and protein sequence using the Transdecoder plugin in Trinity. Reference assemblies were annotated with the Trinotate pipeline to identify GO categories in the Swiss-Prot database (UniProt Consortium, 2015).

Translated files were then used in OrthoVenn (Wang et al., 2015) using default parameters to compare presence/absence for each stage and species, as well as to identify GO categories and any GO enrichments for different stages of development. Differential gene expression analyses were conducted using the EdgeR Bioconductor package (Robinson et al., 2010) for comparison within species at different developmental stages. For *S. australis*, two biological replicates for half (50%), mid (75%), and mature (100%) stages were used with a p-value cut-off of 0.05 and a 4-fold expression change. The small stage (25%) of *S. australis* was not included due to low confidence in one of the assemblies, in which only 13 genes met the 1 fpkm threshold used for filtering. Comparisons of developmental stages in *S. splendens* subsp. *grantii* were conducted in a different fashion due to a high false discovery rate when the preceding method was used. Similar problems in examining fold-changes in genes with low expression have been observed before (Butler et al., 2014). For *S. splendens* subsp. *grantii*, reads for the four stages were pooled prior to the differential expression analysis, resulting in only one input for each stage. This method leads to the inability to perform statistical analyses on the levels of expression; however, it provides evidence of candidate genes to compare to the results found in the other species (Butler et al., 2014). One final comparison was performed using the mature (100%) stages of both individuals of *S. australis* and *S. splendens* subsp. *grantii* to compare expression levels in the final stages of development between species using the reference assembly created using all raw reads. The goal of this last comparison was to investigate any expression differences between previously reported candidate genes.

## Results

### Phylogenetic analysis

Three separate analyses were performed: plastid coding regions only, complete or nearly complete plastome and nuclear markers. For the data set of the complete plastid genome, coverage of the reference plastome ranged from 62.9% (96,715 of 153,853 bp) for *S. caruifolia* to 99.8% for the field accession of *S. splendens* subsp. *splendens* (153,504 of 153,853 bp). Average coverage depth of the plastome ranged from 1.7x to 1379x among samples. For the nuclear genes, the number of loci for each individual ranged from 52 to 90 (Table 1). The plastid coding alignment consisted of 64,500 bp, with 11.5% missing data (cells were coded as either missing if data were lacking or Ns due to low coverage), while the complete plastome alignment had 157,884 bp with 20.2% missing data. For the 90 nuclear genes that had sufficient coverage and overlap between accessions, the concatenated alignment was 147,028 bp with 37.2% missing data.

**Figure 1:**
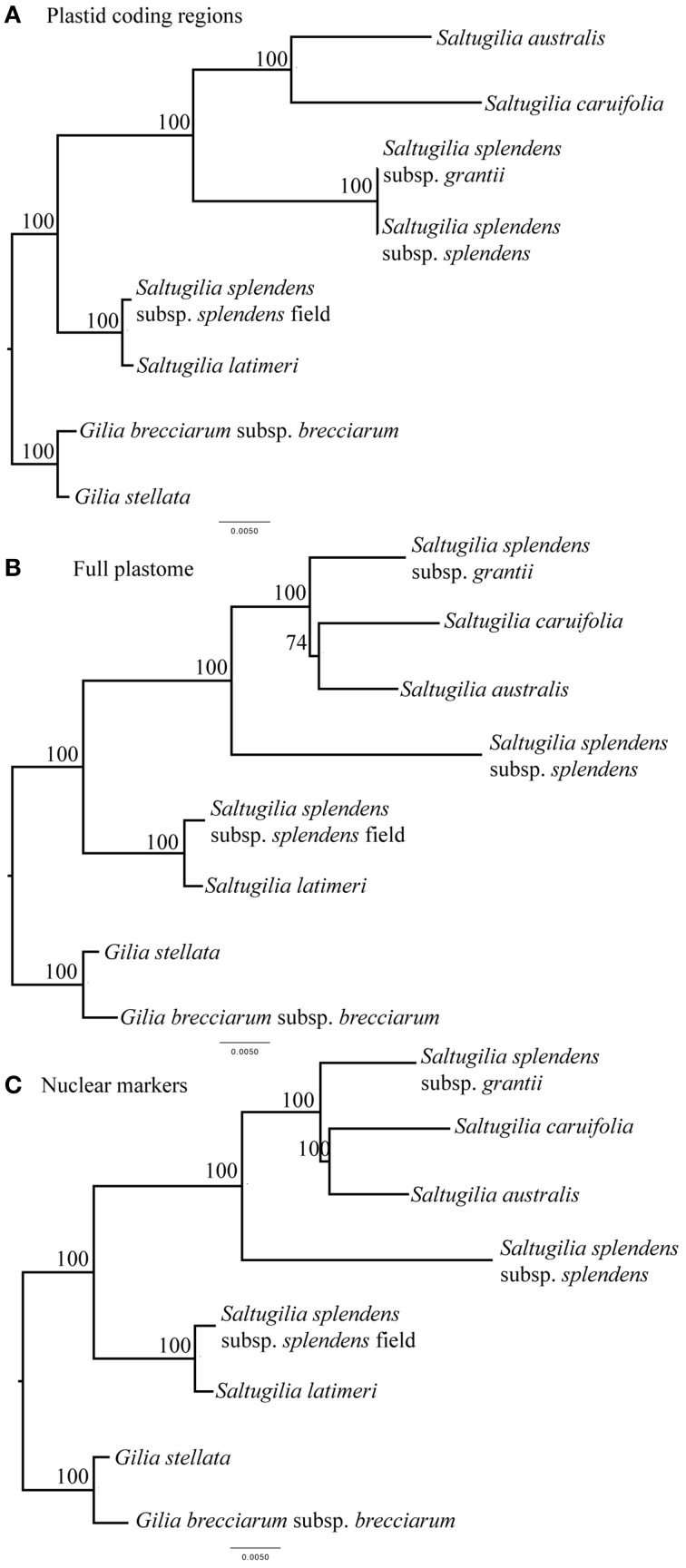
**Phylogenetic relationships of the taxa in ***Saltugilia*** using a maximum-likelihood framework with bootstrap support values shown**. **(A)** concatenated data set comprising plastid coding regions, **(B)** complete plastome sequence, and **(C)** concatenated data set of 90 nuclear loci.

The phylogenetic trees for all three analyses are compared in Figure 1. In the tree based on plastid coding regions, *S. australis* is sister to *S. caruifolia*, with that clade sister to *S. splendens* subsp. *grantii* and *S. splendens* subsp. *splendens*. This four-taxon clade is sister to *S. latimeri* and the field accession of *S. splendens* subsp. *splendens*. Bootstrap support at all nodes is 100%. The trees derived from the complete plastome and the nuclear genes data sets are identical and give a slightly different topology than that from the plastid coding regions, with *S. splendens* subsp. *grantii* sister to *S. australis* and *S. caruifolia*, with *S. splendens* subsp. *splendens* sister to these three taxa. Bootstrap support for all of these nodes is also 100%, except for the node leading to *S. australis* and *S. caruifolia*, which has a bootstrap value of 74% in the complete plastome tree. The relationships of *S. latimeri* and the field accession of *S. splendens* subsp. *splendens* are the same in the trees resulting from all three data sets. The incongruence between the tree derived from the plastid coding genes vs. those of the other two data sets may be the result of a lack of phylogenetic signal in the plastid coding genes. The branch lengths leading to *S. splendens* subsp. *grantii* and *S. splendens* subsp. *splendens* are very short. With the additional data obtained from the complete plastome and nuclear genes, these taxa do not form a clade.

### Morphology

**Figure 2:**
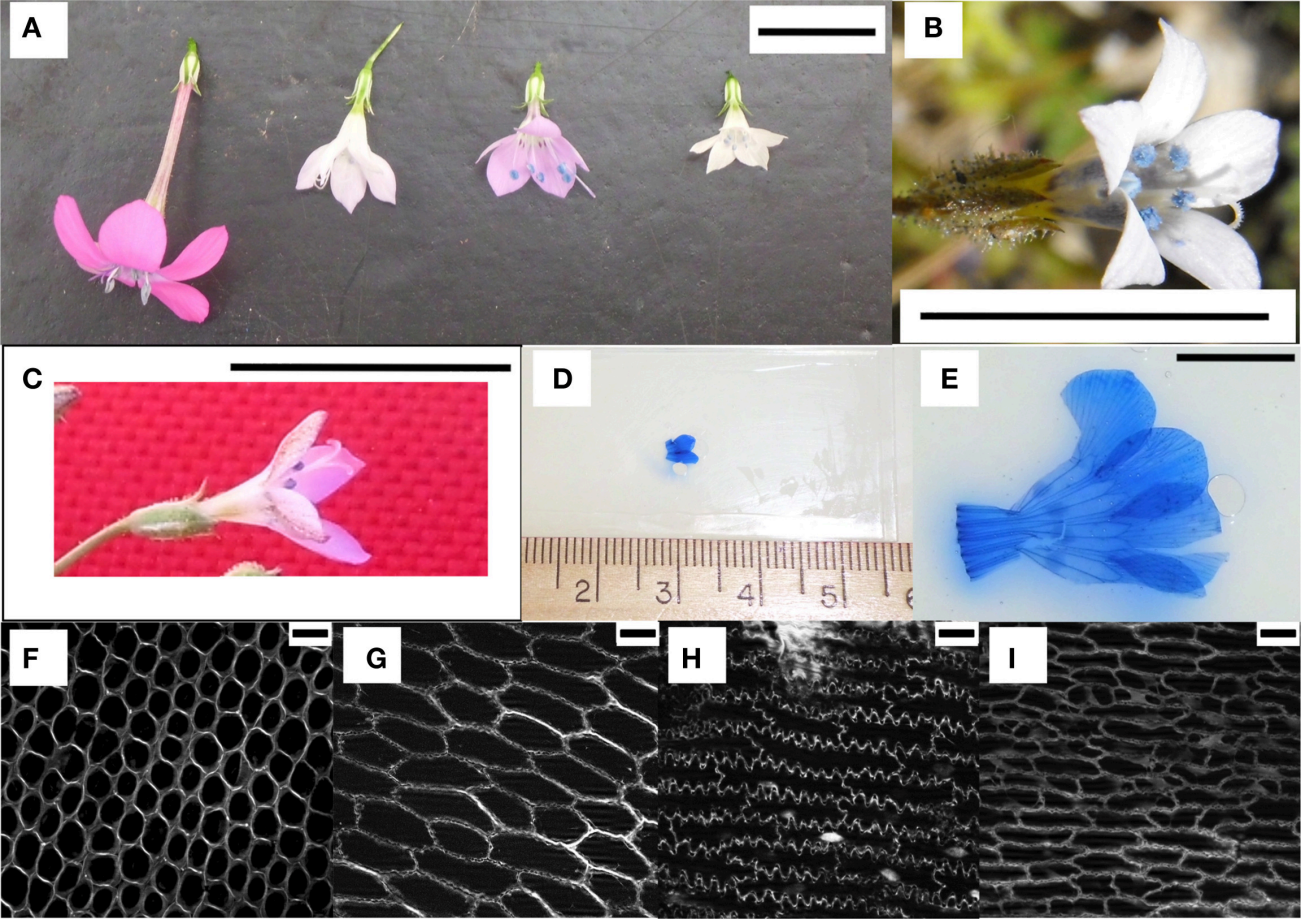
**Examples of flowers and cells visualized. (A)** The four species of *Saltugilia* grown in the greenhouses at the University of Florida. From left to right: *S. splendens* subsp. *grantii, S. splendens* subsp. *splendens, S. caruifolia* and *S. australis*. **(B)** Flower of *Gilia stellata*, one of the species used as an outgroup. **(C)** *S. latimeri* collected in Joshua Tree National Park, CA, USA. **(D)** Prepped microscope slide of the small stage of *S. australis*. **(E)** Prepped microscope slide of the mature stage of *S. splendens* subsp. *grantii*. **(F–I)** Representatives of the four types of cells observed in flower material: conical, transition, jigsaw and elongated cells respectively. Scale bars in **(A–C,E)** are 1 cm, and **(F–I)** are 20 μM.

The flowers of *Saltugilia* range in size from 0.8 to 2.5 cm. The smallest flowers were observed in *S. australis* (0.8–1.1 cm) and *S. latimeri* (0.8–1.0 cm), followed by *S. caruifolia* (1.0–1.2 cm), the greenhouse accession of *S. splendens* subsp. *splendens* (0.9–1.3 cm) and the field accession of *S. splendens* subsp. *splendens* at (1.1–1.5 cm), with the largest flowers belonging to *S. splendens* subsp. *grantii* (2.3–2.5 cm). The two species of *Gilia* both had flowers with corollas of 0.8–0.9 cm (Figures 2A–E).

Four cell types with different shapes were characterized in petal material: conical, transition, elongated and jigsaw cells (Figures 2E–I). The first three cell types were found in all developmental stages (25, 50, 75, and 100%) with jigsaw cells appearing predominantly in mature flowers. Exceptions to this pattern were observed in the field accessions of *S. splendens* subsp. *splendens* and *S. latimeri*, which have jigsaw cells in half, mid and mature flowers, and in the greenhouse accession of *S. splendens* subsp. *splendens*, which lacks elongated cells at the base of the petal tube.

**Figure 3:**
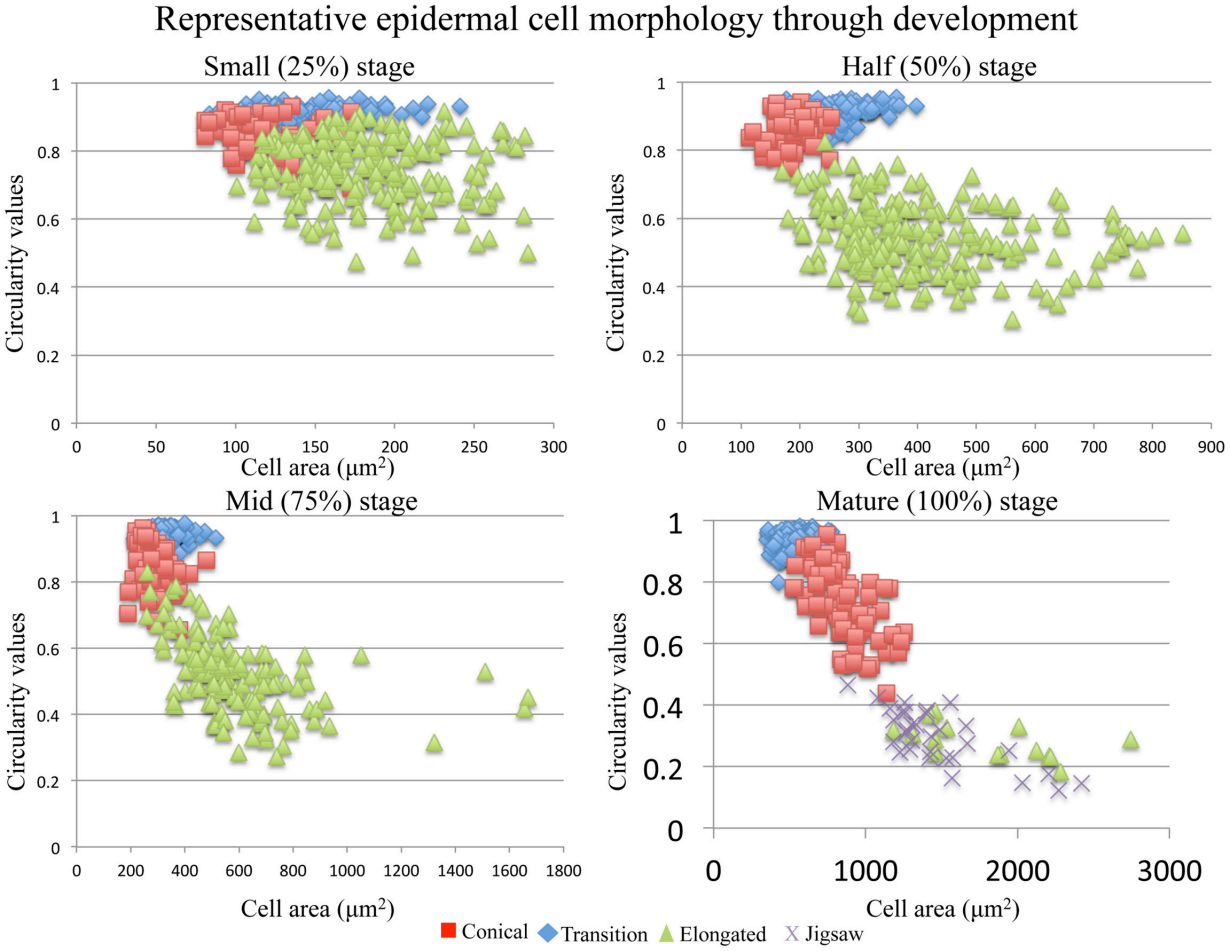
**Representative cell area vs. cell circularity through development of conical, transition, elongated and jigsaw cells**. The closer the circularity value is to 1, the more round the cell.

As the flower develops, conical cells maintain their circular shape with a circularity value ranging from 0.85 to 0.95; however, these cells become larger with a 2.4- to 5.5-fold increase in size. The median observed increase in size is a 3.5-fold increase in cell size between small (25%) and mature flowers of *S. caruifolia, S. splendens* subsp. *grantii, S. splendens* subsp. *splendens* and the field accession of *S. splendens* subsp. *splendens*. The outlier is *S. australis* with a fold change of 5.5 between small (25%) flowers and mature flowers, with both *Gilia* accessions showing the smallest fold change of 2.4 (*G. brecciarum* subsp. *brecciarum*) and 2.5 (*G. stellata*). Along the lobe/tube margin is a distinct cell type defined here as transition cells. These cells are more elongated than conical cells with a circularity value of approximately 0.7 in mature stages, although in earlier stages the circularity values are sometimes very close to those of conical cells (Figure 3). In developing flowers, the corolla tube is composed of elongated cells with a circularity value of 0.7 in small (25%) flowers, then becoming more elongated toward the base of the corolla tube, with circularity values declining in the half stage (0.6) and mid stage (0.5) and a circularity value of 0.2–0.4 in mature flowers. The average size of these cells increased by 5.2- to 9.6-fold between small and mature flowers, with the largest observed change in *S. splendens* subsp. *grantii* (Supplemental Table 2). In mature flowers, the corolla tube is composed of a second type of cells called jigsaw cells. These cells have a circularity of 0.2–0.3 and often have a slightly smaller area than elongated cells at the same developmental stage (Supplemental Figure 1).

**Figure 4:**
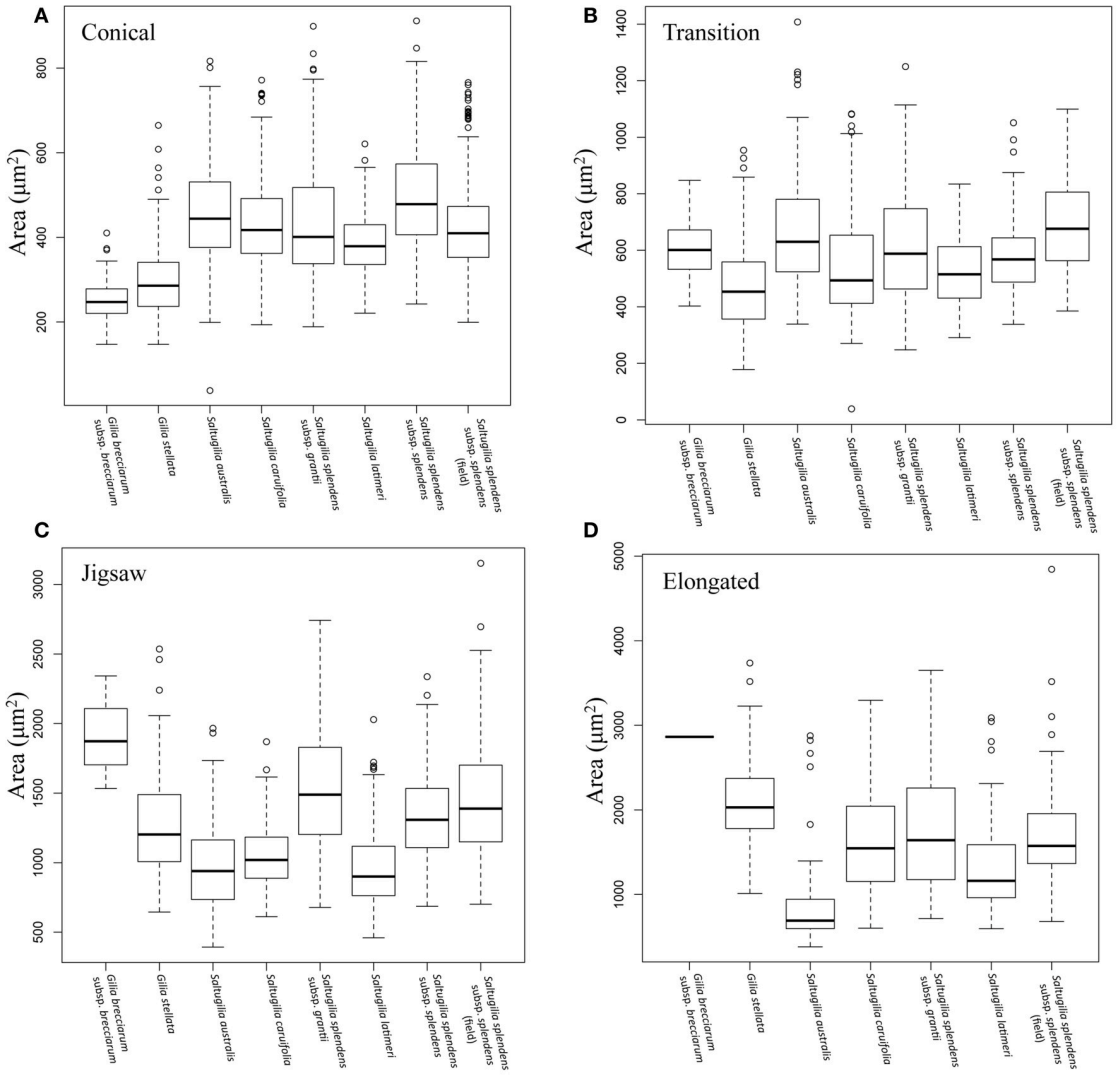
**Box plots comparing the area of each cell type in mature flowers of all species. (A)** Mature conical cells, **(B)** mature transition cells, **(C)** mature jigsaw cells, **(D)** mature elongated cells.

As the flower develops, later stages of development have statistically larger cells than earlier developmental stages of the same cell type in all cases except in transition cells (ANOVA; *F* = 542.6, *p* < 0.001; Supplemental Figure 1). In *S. latimeri* and the field accession of *S. splendens* subsp. *splendens*, the transition cells between the half stage (50%) and the mid stage (75%) are not significantly different in size. In all taxa, the conical and transition cells do not differ significantly in size during the early developmental stages. Comparison of cell size between species was conducted for all four stages, with the mature comparisons visualized in Figure 4.

Conical cells in mature flowers overlap in size across the six accessions of *Saltugilia*, but are significantly different in size (*F* = 128.8, *p* < 0.001) except for cells of *S. splendens* subsp. *grantii*, which are not significantly different from *S. australis* (*p* = 0.128), *S. caruifolia* (*p* = 0.999) and the field accession of *S. splendens* subsp. *splendens* (*p* = 0.807). Jigsaw cells show two distinct categories: the larger cells of *S. splendens* subsp. *grantii* and both accessions of *S. splendens* subsp. *splendens*, and the smaller cells found in *S. australis, S. caruifolia* and *S. latimeri* (*F* = 38.79, *p* < 0.001). The elongated cells of *S. caruifolia, S. splendens* subsp. *grantii* and the field accession *S. splendens* subsp. *splendens* are all similar in size, while the cells of *S. australis* are smaller (*F* = 18.52, *p* = 0.001). The elongated cells of *S. latimeri* are intermediate in size, being significantly larger than *S. australis* (*p* < 0.001) and significantly smaller than the field accession of *S. splendens* subsp. *splendens* (*p* = 0.005). However, no significant differences between the other comparisons of elongated cells were observed.

**Figure 5:**
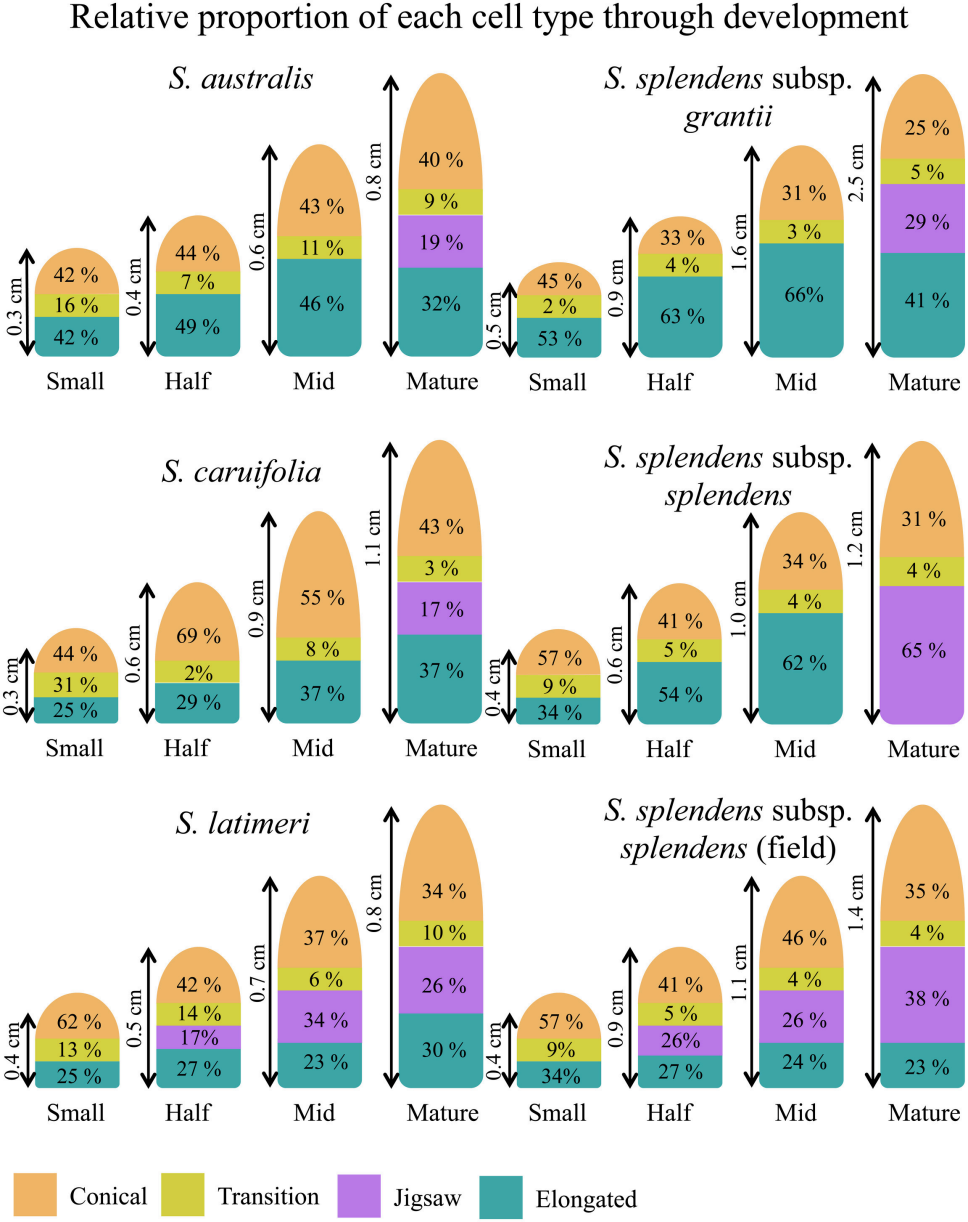
**Proportions of cell types through the four stages of development for all taxa investigated in one plant of each taxon**.

The relative abundance of each cell type changes across developmental stages, as well as among species (Figure 5). In early stages of development (small, 25%), conical cells make up roughly half of the total flower length, between 41.6 and 62.5%. As the flowers develop, the proportion of the flower composed of conical cells decreases to 25–42.8%, while the length of the corolla tube composed of conical cells increases. In *S. splendens* subsp. *splendens*, the proportion of the corolla composed of conical cells decreases from 57.1 to 31.3% through development, while the length of the corolla composed of conical cells increases from 0.23 to 0.38 cm. In general, the proportion of the corolla composed of elongated cells increases from the small to mid developmental stages, but then decreases substantially with the formation of jigsaw cells predominantly in mature flowers (Supplemental Table 3). The two field-collected samples, *S. latimeri* and *S. splendens* subsp. *splendens*, both exhibit the formation of jigsaw cells starting at the half stage of development. *Gilia stellata* was also collected in the field, but does not exhibit jigsaw cells in any stage except the mature stage.

**Figure 6:**
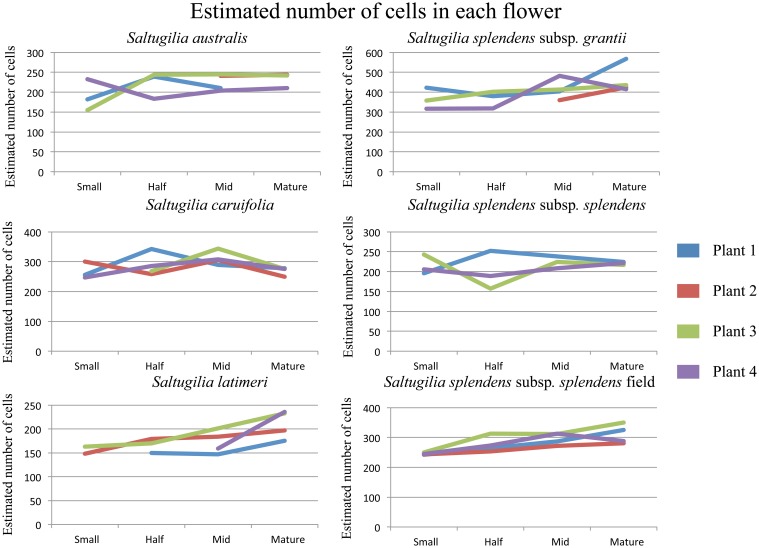
**Estimated cell counts for each of the four individual plants for each taxon of ***Saltugilia*****. Number of cells was estimated by using the mean bounding box width for each cell type through flower development.

Estimates of total cell numbers, as well as the number of each cell type, were computed using the mean width of the bounding box around each measured cell (Supplemental Table 4). Only *S. splendens* subsp. *grantii* and the field accession of *S. splendens* subsp. *splendens* show increased numbers of cells between stages of development (Figure 6). These taxa have roughly 100 more cells in mature stages than at the small developmental stage. Estimates of the number of cells in mature flowers of the two *Gilia* species were similar, with a maximum of 219 cells in *G. brecciarum* subsp. *brecciarum* and 213 in *G. stellata*. The small-flowered species of *Saltugilia* have similar estimated maximum cell numbers, 245 cells in *S. australis*, 236 in *S. latimeri* and 252 in *S. splendens* subsp. *splendens*. *Saltugilia caruifolia* has a maximum estimate of 344 cells, and the two taxa with the largest flowers, the field accession of *S. splendens* subsp. *splendens* and *S. splendens* subsp. *grantii*, have 350 and 567 cells, respectively.

### Transcriptome analysis

The filtered reference transcriptome of *S. australis* consists of 126,343 transcripts composed of 77,498,390 bases with a GC content of 44.7% and an n50 of 802 bp. The filtered reference for *S. splendens* subsp. *grantii* consists of 160,429 transcripts composed of 95,491,059 bases with a GC content of 44.5% and an n50 of 794 bp. The BLAST analysis of the *S. australis* transcripts against the SwissProt protein database yielded hits for 6128 transcripts covering at least 80% of the protein lengths of 15,136 unique known proteins. A BLAST analysis using the *S. splendens* subsp. *grantii* transcripts against SwissProt resulted in 5988 hits covering at least 80% of the protein lengths of 15,426 unique proteins. Functional annotation of gene ontology (GO) categories was also investigated for transcripts of all three reference transcriptomes. For *S. australis*, 41,874 transcripts were annotated with GO categories, whereas the *S. splendens* subsp. *grantii* transcriptome has 50,174 transcripts with GO category annotations. The combined reference, with all reads, has 62,253 transcripts annotated with GO categories (Supplemental Table 5).

**Figure 7:**
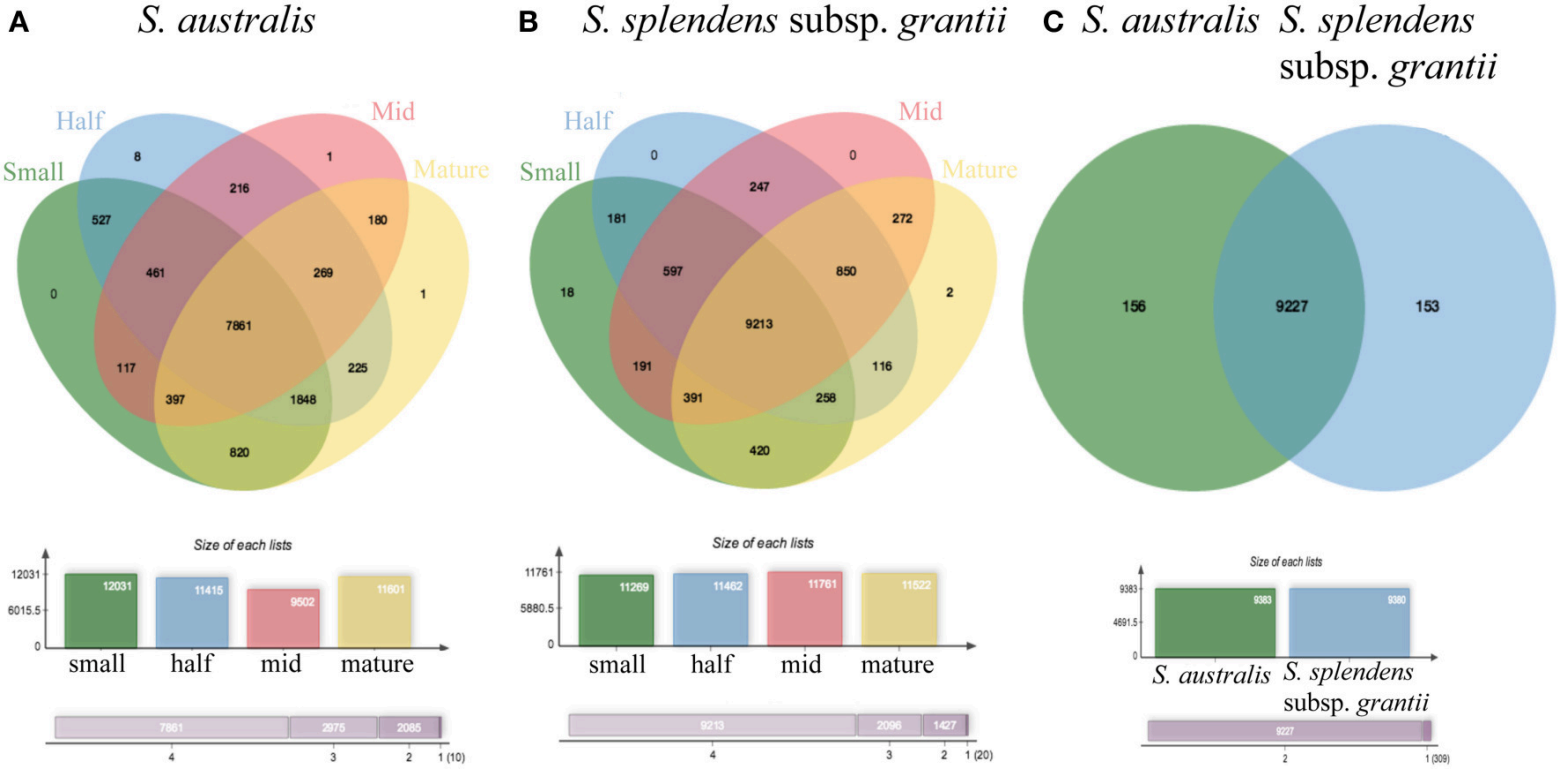
**OrthoVenn diagrams of pooled reads for (A) each stage of development: small, half, mid and mature for ***S. australis***, (B) each stage of development: small, half, mid and mature for ***S. splendens*** subsp. ***grantii***, and (C) ***S. australis*** and ***S. splendens*** subsp. ***grantii*** showing clusters of proteins that are shared and unique to each species**.

Comparing the two reference transcriptomes using OrthoVenn, *S. australis* has 14,682 proteins in 9375 clusters, and *S. splendens* subsp. *grantii* has 14,545 proteins in 9362 clusters (Figure 7C). In all, 14,305 proteins in 9229 clusters are shared between the two taxa, with an additional 146 clusters unique to *S. australis* and 133 clusters unique to *S. splendens* subsp. *grantii*. The five GO categories with the largest numbers of proteins shared between the two taxa are biological process (2501 proteins), metabolic process (2073 proteins), cellular process (1814 proteins), cellular metabolic process (1743 proteins) and response to stimulus (1228 proteins). Other clusters of proteins that might be relevant for investigating differences in flower size are developmental process (708 proteins), growth (149 proteins), cell wall organization (141 proteins), cell growth (112 proteins), cell division (80 proteins) and cell proliferation (28 proteins).

Of the protein clusters unique to *S. australis*, the top five GO categories are biological process (50 proteins), metabolic process (42 proteins), cellular process (39 proteins), cellular metabolic process (38 proteins) and nitrogen compound metabolic process (30 proteins). Additionally, 11 proteins are associated with developmental process, six with cell cycle, three with cell division, three with growth, two with cell proliferation, two with cell wall organization and two with cell growth. Categories that appear to be enriched only in *S. australis* are ammonium transmembrane transporter activity and DNA topoisomerase activity, both associated with the molecular function category. The top five protein clusters unique to *S. splendens* subsp. *grantii* are the same top five clusters shared between the two species. The only GO category enriched only in *S. splendens* subsp. *grantii* is maintenance of seed dormancy, a biological process.

Within each species, the number of protein clusters varies across developmental stages. In *S. australis*, the four stages exhibit 12,031 (small stage), 11,415 (half stage), 9502 (mid stage), and 11,601 (mature stage) protein clusters (Figure 7A). Overall, 7861 protein clusters are shared across the four developmental stages. The small stage has no unique clusters compared to the other stages, whereas the half stage has eight, and the mid and mature stages each have one unique protein cluster. The unique clusters in the half stage are the only clusters that have significantly enriched GO terms, and those are: polysaccharide binding, megasporogenesis and radial pattern formation. Most clusters shared between at least two stages of development in *S. australis* do not have any enriched GO terms except oligopeptide transporter activity shared between the half and mid stage, phosphatidylinositol-4-phosophate binding shared between the small and mid stages and plasma membrane ATP synthesis coupled proton transport shared between the small and half stages.

In *S. splendens* subsp. *grantii*, the number of protein clusters is very similar across stages, with 11,377 (small stage), 11,491 (half stage), 11,781 (mid stage), and 11,505 (mature stage) clusters. Most of these protein clusters (9198) are shared across all stages (Figure 7B). The small stage has the most unique clusters with eight, while the mid stage has one and the mature stage has two, with the half stage having no unique clusters. The unique clusters of the small-stage flowers have GO enrichment categories of regulation of growth rate and positive regulator of organ growth, while the unique cluster in the mid stage is enriched with the GO cellular component term nuclear speck. The unique clusters found in the mature stage are enriched for four terms: ATP synthesis coupled electron transport, phosphorelay signal transduction system, NADH dehydrogenase activity and respiratory chain. As seen in *S. australis*, most shared clusters do not have any GO-enriched terms except in a few specific cases. The clusters shared between small and half stages are enriched for condensing complex, while the clusters shared between the small, half and mid stages are enriched for cell wall modification involved in multidimensional cell growth. There are four terms enriched in the clusters shared between the small and mature stages: sulfate transmembrane-transporting ATPase activity, valine-tRNA ligase activity, aminoacyl-tRNA editing activity and valyl-tRNA aminoacylation. Four additional terms are enriched in the clusters shared between the mid and mature stages: regulation of development (heterochronic), gene silencing by miRNA, histone deacetylation and histone deacetylase activity.

**Figure 8:**
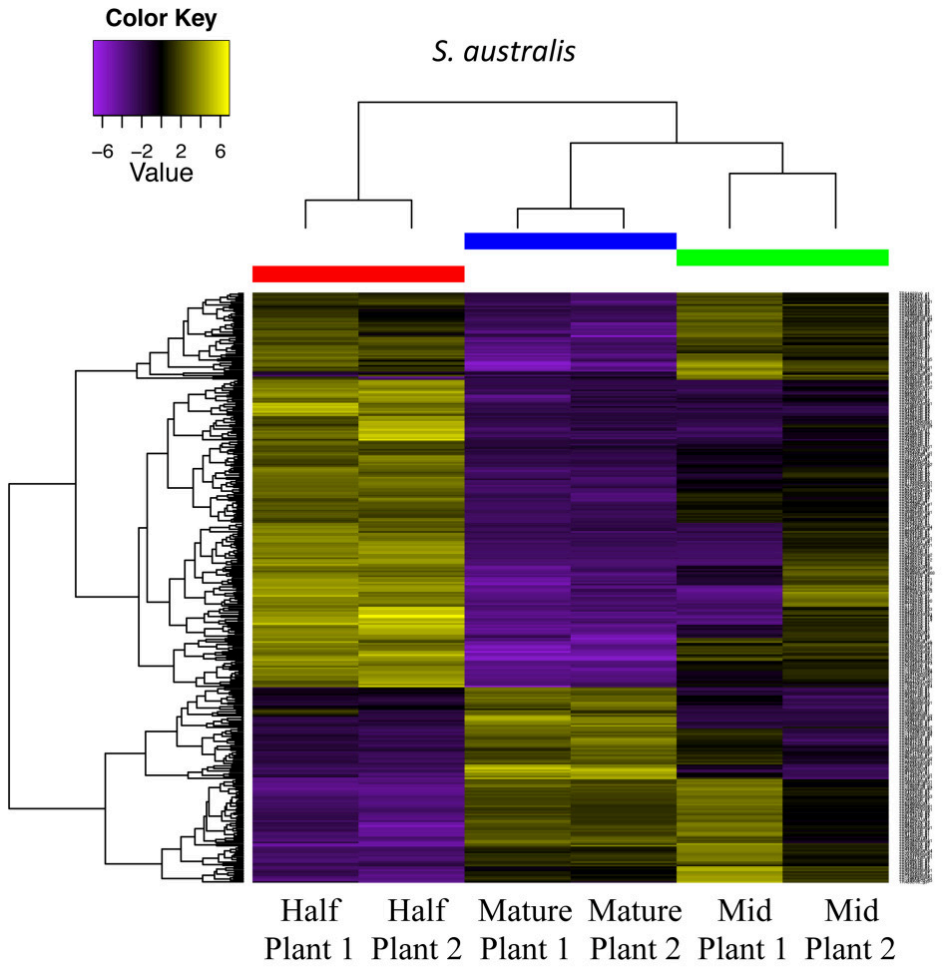
**Differential gene expression analysis among half, mid and mature stages of development for two plants of ***S. australis*****. Cutoff for differentially expressed genes was a 4-fold change in expression levels with a *p*-value of 0.05. Genes colored in yellow are up-regulated and genes colored purple are down-regulated compared to the other stages.

In *S. australis*, the greatest shift in differential expression occurs between the half and mature stages, in which 300 transcripts are differentially expressed, with 208 transcripts up-regulated in mature flowers (Figure 8; Table 2). Of these up-regulated transcripts, 133 are cellular component genes and seven molecular function genes. Of the cellular component transcripts, four are associated with the cell wall. The comparison between mature and mid stages resulted in 95 differentially expressed transcripts, with 67 of those up-regulated in the mid stage and 28 up-regulated in the mature stage. In comparison, only 43 transcripts are differentially expressed between the half and mid stages. (Comparisons involving flowers in small stages were not conducted due to lack of replication, with one of the transcriptomes removed because only 13 transcripts passed the 1 fpkm filtering used.)

**Table 2:** **Results from differential gene expression analysis between stages of ***Saltugilia australis*** (Sa) and ***S. splendens*** subsp. ***grantii*** (Sg)**.

**Comparison**	**Up-regulated transcriptome**	**Biological process**	**Cellular component**	**Molecular function**	**Total**
Sa Mature vs. Sg Mature	Sa Mature	4	130	41	382
	Sg Mature	1	133	32	354
Sa Mature vs. Sa Half	Sa Mature	0	133	7	208
	Sa Half	1	46	15	92
Sa Half vs. Sa Mid	Sa Half	0	1	1	4
	Sa Mid	0	22	3	39
Sa Mature vs. Sa Mid	Sa Mature	1	13	7	28
	Sa Mid	1	31	4	67
Sg Mature vs. Sg Half	Sg Mature	2	176	77	427
	Sg Half	7	263	40	452
Sg Mature vs. Sg Mid	Sg Mature	2	184	98	506
	Sg Mid	4	166	40	280
Sg Mature vs. Sg Small	Sg Mature	3	220	44	474
	Sg Small	7	286	38	526
Sg Half vs. Sg Mid	Sg Half	0	8	1	19
	Sg Mid	0	12	5	27
Sg Half vs. Sg Small	Sg Half	6	375	65	743
	Sg Small	4	306	123	713
Sg Mid vs. Sg Small	Sg Mid	6	411	91	819
	Sg Small	5	457	160	1017

*GO categories for the up-regulated transcripts from comparison are listed, as well as the total number of up-regualated transcripts in each transcriptome*.

In the large-flowered *S. splendens* subsp. *grantii*, in constrast, the greatest shift in expression occurs between the small to mid stages of flower development, with 1836 transcripts exhibiting potential differential expression, with 819 being up-regulated in mid stage flowers and 1017 up-regulated in small stage flowers. The second greatest shift in expression occurs between small to half stages of flower development with 1456 transcripts showing differential expression. In the transition between mid and mature flowers, the number of transcripts with differential expression in *S. splendens* subsp. *grantii* is nearly 8-fold greater than in the small-flowered *S. australis*, with 786 transcripts showing differential expression, 506 of which are up-regulated in mature flowers (compared with only 28 in *S. australis*). Of these, 184 transcripts represent cellular component genes, 98 molecular function genes and two biological function genes. Twelve of the cellular component genes are associated with the cell wall, including cell wall organization, growth polysaccharide catabolic process and trehalose metabolic process. One of the molecular function genes is associated with the cell wall: polysaccharide biosynthetic process. The two biological process genes that are up-regulated in the mature flower both involve cell division. When comparing mature flowers to half-stage flowers, *S. splendens* subsp. *grantii* (879 transcripts) showed a 4.2-fold increase in the number of differentially expressed transcripts compared to *S. australis* (300 transcripts). Similar to *S. australis*, the comparison of mid and half stages resulted in the fewest number of differentially expressed transcripts with 46.

**Figure 9:**
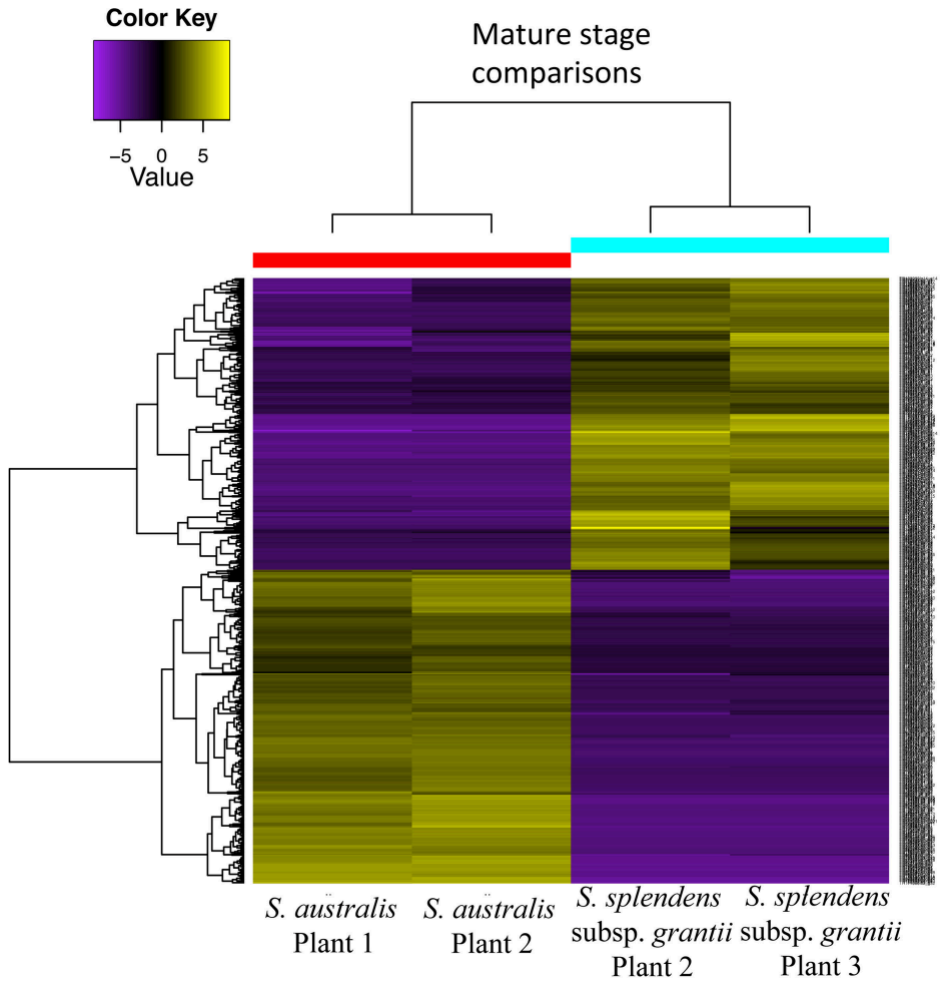
**Differential gene expression analysis between mature stages of development for two plants of ***S. australis*** and ***S. splendens*** subsp. ***grantii*****. Cutoff for differentially expressed genes was a 4-fold change in expression levels with a *p*-value of 0.05. Genes colored in yellow are up-regulated and genes colored purple are down-regulated compared to the other species.

Comparison between mature stages of *S. australis* and *S. splendens* subsp. *grantii* yields a total of 736 differentially expressed transcripts (Figure 9). Of these, 382 are up-regulated in *S. australis*, with 354 transcripts up-regulated in *S. splendens* subsp. *grantii* (Supplemental Table 6). Of the up-regulated transcripts in *S. australis*, 175 produce BLAST annotations to GO categories, with 130 of these representing cellular component genes, 41 molecular function genes and four biological processes. Of the cellular component GO category transcripts, 15 represent transcripts associated with the cell wall, including specific associations with the vacuole and the regulation of cell proliferation. Three of the molecular function transcripts have GO categories associated with organization of the cell wall, with an additional four transcripts involved in pectin catabolic process. The four biological process category transcripts involve the auxin activated signaling pathway and responses to biotic stimulus and stress. Of the up-regulated transcripts in *S. splendens* subsp. *grantii*, 166 BLAST to known GO categories, with 133 of those cellular component genes, 32 molecular function genes and one biological process. Twenty-one of the cellular component genes are involved with cell wall formation, including carbohydrate metabolic process, pectin catabolic process and cell wall organization, with one of the molecular function genes associated with the cell wall and cell wall organization.

### Phylogenetic integration

**Figure 10:**
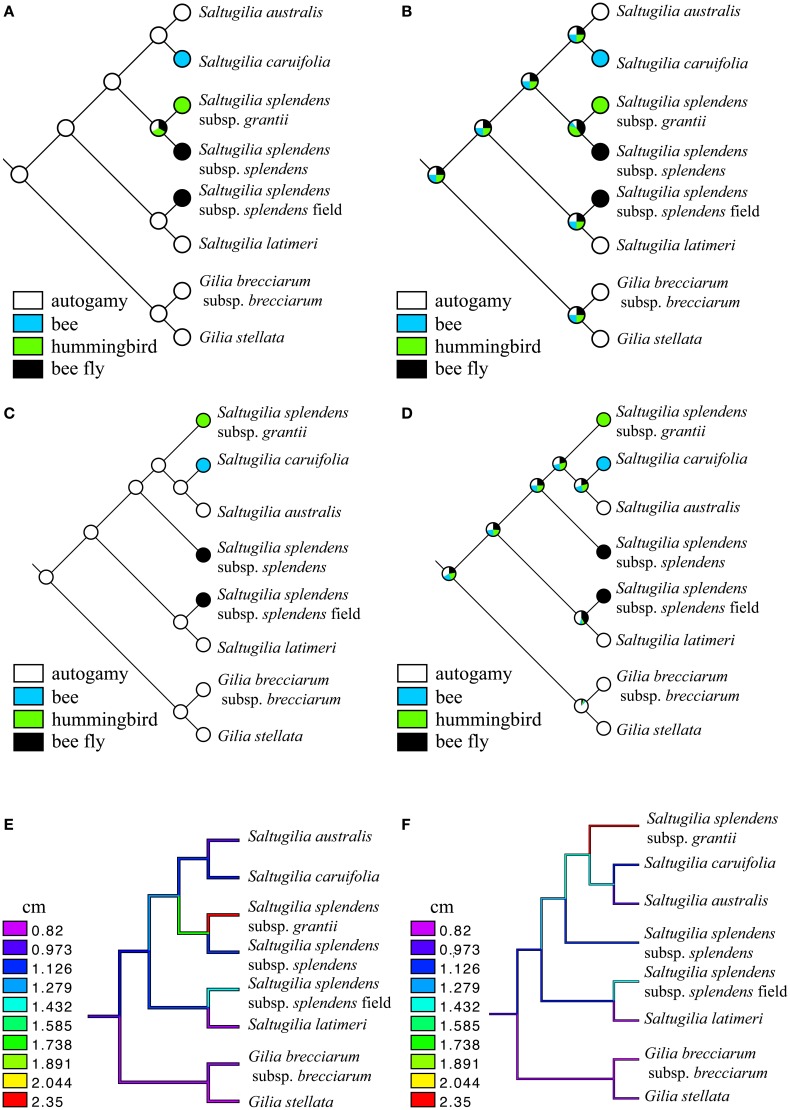
**Ancestral-state reconstructions of pollinators (A–D) and size (E,F) under a maximum parsimony (MP) and maximum-likelihood (ML) framework using the two competing topologies. (A)** MP of pollinators using the topology from plastid coding genes, **(B)** ML of pollinators using the topology from plastid coding genes, **(C)** MP of pollinators using the topology from the complete plastome and nuclear genes, **(D)** ML of pollinators using the topology from the complete plastome and nuclear genes, **(E)** continuous reconstruction of flower size using the topology from plastid coding genes, and **(F)** continuous reconstruction of flower size using the topology from the complete plastome and nuclear genes.

Integrating the phylogeny of *Saltugilia* with the flower size data shows two independent shifts to larger corollas using either topology recovered (Figure 10). Using the topology based on the coding regions of the plastome, one case of elongation involves the clade of *S. splendens* subsp. *grantii* and *S. splendens* subsp. *splendens*, with respective sizes of 2.3–2.5 cm and 0.9–1.3 cm. The second transition is the field accession of *S. splendens* subsp. *splendens*, which has corollas that range in size from 1.1 to 1.5 cm. When using the complete plastome topology and nuclear genes, the same transitions are evident for *S. splendens* subsp. *grantii* and the field accession of *S. splendens* subsp. *splendens*. The difference between the reconstructions is the corolla size inferred for some of the internal nodes.

Multiple shifts in pollinators are evident. When using MP, regardless of topology, the putative ancestor of *Saltugilia* is reconstructed as autogamous. Subsequent transitions to bee, hummingbird and bee fly (two transitions) are evident. Under an ML framework, the evolutionary trajectory is not as clear. In both topologies, the ancestral state for *Saltugilia* is equivocal, with equal likelihoods for each of the pollinator types, with subsequent transitions therefore difficult to determine.

There is also phylogenetic signal in cell type. Both *S. latimeri* and the field sample of *S. splendens* subsp. *splendens* possess jigsaw cells in the corolla during early stages of development, whereas all other taxa only possess these cells in mature flowers. These taxa are sisters in both topologies, and there appears to be a single evolutionary transition yielding jigsaw cells early in development.

## Discussion

### Phylogenetic relationships in *Saltugilia*

Previous phylogenetic analyses have included multiple members of *Saltugilia*, but those either lacked some taxa or were based on only a small number of markers (Weese and Johnson, 2005; Johnson, 2007; Landis et al., in review), and not all relationships were highly supported. In this study, analyses of three data sets—the coding regions of the plastome, the complete plastome and 90 nuclear loci—yield high bootstrap values for all nodes. This concatenation approach for the coding regions of the plastome and the complete plastome has been utilized before in previous studies given that the plastome is inherited as a single locus (Small et al., 1998; Parks et al., 2009; Moore et al., 2010; Ruhfel et al., 2014). However, even though unlinked nuclear loci may have different coalescence histories, several recent studies have found that concatenation methods yield results similar to species tree reconciliation methods (e.g., Thompson et al., 2014; Tonini et al., 2015), supporting our use of a concatenated data set for the nuclear markers as well.

All three datasets suggest the same phylogenetic relatedness of species, except for the placement of the *S. splendens* taxa. If the topology based on both the complete plastome and nuclear genes is correct, taxonomic revisions are warranted, given the lack of monophyly of the accessions of *S. splendens*. Recognition of each accession as representing a distinct species would be consistent with both the phylogeny and the morphology of the three accessions, but further work is needed to investigate these taxa. In earlier phylogenetic analyses, *S. latimeri* was found to be sister to *S. australis* (Weese and Johnson, 2005; Johnson, 2007). However, these analyses were based on only one to five genes, and usually a combination of nuclear and plastid genes, which may show topological incongruence due to hybridization (Soltis and Kuzoff, 1995; Sang et al., 1997; Okuyama et al., 2005). Our analysis does not show this relationship between *S. latimeri* and *S. australis*, however, as we recovered *S. latimeri* to be sister to the field accession of *S. splendens* subsp. *splendens*. This result suggests that further phylogenetic and taxonomic work is needed, particularly because of high levels of morphological variability in *S. splendens*, making identification of subspecies difficult (Mark Porter, personal communication). *Gilia stellata* was deemed an appropriate outgroup because it has long been associated with *Saltugilia* taxonomically, with the proposal that it be transferred to *Saltugilia* (Grant, 1959; Grant and Grant, 1965), but sufficient evidence for this move has never been provided. Our analysis shows that it is sister to *G. brecciarum* based on both plastome and nuclear data, supporting its placement in *Gilia*.

Transitions from outcrossing to selfing are prevalent in many angiosperm groups (e.g., Stebbins, 1974; Bull and Charnov, 1985; Schoen et al., 1996; Barrett, 2003, 2008, 2013). In transitions involving changes in outcrossing vector, the most numerous transitions are bee to hummingbird pollination, with the reverse being observed less frequently (Barrett, 2013). Previous analysis of pollinators in Polemoniaceae, with sampling of nearly all species in the family, shows that the ancestor of *Saltugilia* was likely bee-pollinated (Landis et al., in review). If this is indeed the case, then with the increased sampling of *Saltugilia* in the present study, we observe two independent transitions to autogamy (*S. australis* and *S. latimeri*), one transition to hummingbird pollination (*S. splendens* subsp. *grantii*) and at least one (possibly two) transition to bee fly pollination (*S. splendens* subsp. *splendens*). However, with only *Saltugilia* and two species of *Gilia* represented in the current study, we reconstruct the ancestor of *Saltugilia* to be autogamous, with gains of bee, hummingbird and bee fly pollination. This discrepancy may be attributed to taxon sampling, not in *Saltugilia* itself, but in its close relatives. Based on the larger analysis in Landis et al. (in review), the sister group to *Saltugilia* is a clade consisting of *Gilia, Collomia*, and *Navarretia*. In both analyses, the ancestor of *Gilia* is reconstructed as autogamous, but in the larger study, the combination of states results in the common ancestor of *Saltugilia* being bee-pollinated and here, autogamous. This highlights the importance of sufficient taxon sampling for character state reconstructions; otherwise, the reconstructions may be skewed to favor only those taxa sampled and not the best overall evolutionary hypothesis.

The importance of taxon sampling does not seem to be as significant when reconstructing flower size within *Saltugilia*. In the large analysis of Polemoniaceae (Landis et al., in review), the common ancestors of both *Saltugilia* and of *Gilia* were reconstructed as having flowers 0.8–1.2 cm in length. In both reconstructions of flower size in the current study, the common ancestor of *Saltugilia* had flowers of 0.97–1.13 cm in length. This concordance between the large and focused analyses may be due to the fact that the entire clade of *Saltugilia, Gilia, Collomia* and *Navarretia* is composed of relatively small flowers (Landis et al., in review), so there are no large outliers that affect the reconstruction of ancestral states. In investigations of flower size, especially in relation to shifts in pollinators, transitions from outcrossing to selfing are often accompanied by transitions to smaller flowers, with examples observed in *Aquilegia paui* (Ranunculaceae; Martinell et al., 2011), *Arabis alpha* (Brassicaceae; Tedder et al., 2015) and *Camissoniopsis cheiranthifolia* (Onagraceae; Button et al., 2012). Our analyses of *Saltugilia* show that selfing species have smaller flowers than their outcrossing congeners, generally due to larger jigsaw and elongated cells comprising the corolla tube in many of the outcrossing species and twice as many cells in the large flowers of the outcrossers as in the small flowers of the selfers.

### Cell morphology and cell size

Four types of cells have been identified in developing flowers of species of *Saltugilia*: conical, transition, elongated and jigsaw cells. Jigsaw cells are evident mostly in mature flowers, but they appear at earlier stages in *S. latimeri* and *S. splendens* subsp. *splendens*. These jigsaw cells are similar in shape to the jigsaw pavement cells observed in leaves (Fu et al., 2009). Previous analyses of epidermal cell shape suggest that these cells are similar to multiple papillate cells (Kay et al., 1981) and tabular rugose cells (Ojeda et al., 2009). The development of leaf jigsaw pavement cells is fairly well characterized (Fu et al., 2005) and thought to be regulated by cell-cell signaling. The jigsaw pavement cells in leaves begin as circular cells and then become elongated before becoming jigsaw shaped (Fu et al., 2005). The cells in the corolla tube of flowers in Polemoniaceae exhibit similar developmental characteristics to these jigsaw pavement cells by starting out more circular in earlier stages, with later elongation and finally formation of jigsaw cells (Figure 3). However, jigsaw cells are not ubiquitous in corolla tubes across angiosperms, as they have not been identified in multiple species of *Petunia* (*P. axillaris, P. exserta*, and *P. integrifolia*; Landis, unpublished data) but have been reported in the flowers of 13 families (Kay et al., 1981), including Caryophyllaceae, Onagraceae, Polygonaceae and Primulaceae, and are thought to be important for rapid petal expansion.

The shape of petal epidermal cells represents an evolutionarily labile trait, hypothesized to be regulated by a small subfamily of duplicate transcription factors (Glover et al., 2015). The petal epidermis, especially the epidermis located in the petal lobes, is composed of conical cells that enhance the attractiveness of the flower to potential pollinators by modifying flower color by focusing light on pigmented cells, affecting surface texture for pollinator grip and affecting floral surface temperature (Kevan and Lane, 1985; Noda et al., 1994; Comba et al., 2000; Whitney and Glover, 2007). In *Antirrhinum*, formation of these conical cells is regulated by a MYB-related transcription factor, *MIXTA*, and related genes *MIXTA-LIKE 1, MIXTA-LIKE 2*, and *MIXTA-LIKE3* (Noda et al., 1994; Glover et al., 1998; Martin et al., 2002; Perez-Rodriguez, 2005; Baumann et al., 2007). Phylogenetic analyses by Brockington et al. (2013) of the closely related *MIXTA* MYB transcription factors showed multiple duplication events, including in the common ancestor of the eudicots and multiple lineage-specific duplications. This pattern of gene duplication and functional diversification suggests that changes in petal epidermal cells involving cell types other than conical cells may likewise be affected by similar patterns of divergence in transcription factors associated with gene duplication events.

In *Saltugilia*, the difference in flower size appears to be determined more by cell size than cell number, although cell numbers also increase in the large flowers in the genus. Given that organ sculpting via localized cell division is the key factor determining the initiation of nectar spurs in *Aquilegia* (Yant et al., 2015), with cell elongation responsible for variation in length of the spurs (Puzey et al., 2012), corolla development, in both shape and size, may involve multiple processes. More specifically, even the size of different parts of the flower (and/or leaves) may be controlled by different mechanisms. For example, in *Petunia*, the area of single cells begins to increase at the base of the petal tube and then gradually progresses toward the tip of the petal lobes, with distinct differences in cell growth and expansion in the bottom third of the corolla (Reale et al., 2002; Stuurman et al., 2004). Our results for *Saltugilia* follow this general pattern. In the earliest developmental stage investigated (25% of mature flower length), the sizes of conical, transition and elongated cells are similar. By the half and mid stages, the elongated cells have had a 2–5-fold change in size, while the conical cells have undergone a 1.5–2-fold increase in size.

In addition to these changes in cell size during development of the corolla in *Saltugilia*, the most obvious change in cell number is the appearance of jigsaw cells in most taxa between the mid and mature stages of flower development. With the formation of jigsaw cells, the number of elongated cells diminishes, in some cases between 20 and 140 cells (Supplemental Table 4). This is in stark contrast to development in *Petunia*, which exhibits no appreciable changes in cell number in any domain of the flower throughout development (Stuurman et al., 2004). In *Saltugilia*, changes in the number of conical and transition cells are much less pronounced. The general trend appears to be a marginal increase in the number of conical cells and a decrease in the number of transition cells. When comparing estimates of total cell numbers across all flowers, the majority of flowers appear to be composed of 200–250 cells. In contrast, three of the four plants of the large-flowered *S. splendens* subsp. *grantii* have estimates ranging between 400 and 450 cells, with the fourth plant estimated to have 567 cells in the mature flower. The field-collected accession of *S. splendens* subsp. *splendens* has flowers with more than 300 cells. These two taxa bear the largest flowers in *Saltugilia* and are clearly non-monophyletic, indicating two independent evolutionary origins of large flowers, in both cases accomplished through increased cell number in conjunction with increased cell size.

### Genetics of flower size

The most abundant GO categories detected in analyses of transcripts from both *S. australis* and *S. splendens* subsp. *grantii* are biological process, metabolic process, cellular process, cellular metabolic process and response to stimuli. However, those transcripts that were mostly up-regulated in *S. splendens* subsp. *grantii* relative to the smaller-flowered *S. australis* were cellular component genes, which were ranked 21st in the total number of genes found in the presence/absence comparison between species. Additionally, some molecular function genes and a small subset of biological process genes were also up-regulated in *S. splendens* subsp. *grantii*. With the formation of jigsaw cells, and their apparent importance in contributing to overall corolla length (Figure 5), genes associated with cell wall formation and organization may provide the genetic framework for larger flowers in *Saltugilia*.

Additional evidence supporting this hypothesis for the importance of cellular component genes comes from comparison of GO categories for differentially expressed transcripts in different developmental stages within species. With a single exception (the comparison of half and mid stages in *S. australis*), all comparisons of gene expression show that the largest category of up-regulated transcripts is for the cellular component genes. This trend is most apparent in the size comparisons of *S. splendens* subsp. *grantii*. Comparisons of the small and mid stage transcriptomes resulted in 411 cell component genes up-regulated in the mid stage and 457 up-regulated in the small stage. The large amount at the small stage may be attributed to the formation of new cells and cell walls, while the up-regulation in the mid stage may be the result of elongated cells starting to undergo the necessary changes to become jigsaw cells. Even though the small stage of *S. australis* could not be included in the developmental comparisons, when comparing the mature and mid stages of flower development to the half stage, transcripts attributed to cell component functions are up-regulated in the later stages, with 133 genes up-regulated in the mature stage compared to the half stage and 22 transcripts up-regulated in the mid stage compared to the half stage. The observation that such a large subset of cellular component transcripts, specifically cell wall modification genes, is up-regulated may not be surprising given that approximately 15% of the *Arabidopsis* genome is dedicated to cell wall formation and modification (Carpita et al., 2001). Weiss et al. (2005) reviewed the importance of the process of cell wall deposition and how the cell wall is formed, producing important changes in floral organ size. Thus, the many genes involved in cell wall formation in general provide a large pool of genes for which expression may be modified to yield variation in cell size and cell number, effecting changes in flower size as well as other traits.

Many published studies have shown possible effects of candidate genes for floral size identified in the model systems *Arabidopsis thaliana* and *Antirrhinum majus* (Herzog et al., 1995; Elliott et al., 1996; Mizukami, 2001; Kim et al., 2002; Disch et al., 2006; Krizek, 2009; Xu and Li, 2011). However, none of the genes hypothesized to play a major role in overall size appears to be differentially expressed between the larger flowers of *S. splendens* subsp. *grantii* and the smaller flowers of *S. australis*. Orthologs of *JAGGED, OPR3, BIG BROTHER, KLH, GAST1* and *GASA* were identified in the reference assemblies for both species and were also detected in each of the developmental stages for both species. These candidate genes are expressed, but not differentially so, between the small-flowered *S. australis* and the large-flowered *S. splendens* subsp. *grantii*, or differentially expressed among stages within species. Because of this, their function and roles in *Saltugilia* are currently uncertain. Additional genes compiled by Hepworth and Lenhard (2014) have been identified to be associated with growth and expansion of leaves. Many of these additional genes, such as *AUXIN RESPONSE FACTOR2, TARGET-OF-RAPAMAYCIN* and *SPINDLY*, were found in the reference assemblies, but were not identified as genes differentially expressed between the two species or between stages within species. The lack of differential expression of these additional genes may not be surprising, however, given that Anastasiou and Lenhard (2007) found that QTL that affect leaf size and shape are largely distinct from those influencing floral organs, at least in *Arabidopsis* and tomato. Therefore, candidate genes from leaf growth and leaf size may not be good candidates for controlling differences in corolla size. Additional avenues for flower size may be the role of hormones, given that in the non-model species *Gaillardia grandiflora*, corolla growth was accomplished by an increase in gibberellin activity (Koning, 1984).

Transcriptome analysis of additional taxa of *Saltugilia* may contribute to the discovery of the genetic component(s) of flower size differences in *Saltugilia*. Comparing the transcriptomes of *S. latimeri* and the field accession of *S. splendens* subsp. *splendens*, especially at earlier stages of development, will be informative, given that jigsaw cells appear earlier in development in both taxa than in the other taxa investigated. Furthermore, functional analyses (using Virus Induced Gene Silencing) and additional expression studies using qPCR in *Saltugilia*, as well as in model species, are needed to evaluate the possible roles of the candidate genes identified here in determining flower size.

## Conclusions

In *Saltugilia*, there are two independent evolutionary transitions from smaller to longer flowers, possibly driven by selective pressures of hummingbird and bee fly pollination. The longer corollas of *S. splendens* subsp. *grantii* and the field accession of *S. splendens* subsp. *splendens* show significant increases in the cell area of jigsaw cells, as well as slightly longer elongated cells. In addition to increased cell size in *S. splendens* subsp. *grantii*, there is also a large increase in the estimated number of cells making up the corolla tube, especially of jigsaw cells. Comparisons of the transcriptome profiles across developmental stages of the taxa having the smallest and largest flowers, including within- and between-taxon comparisons, resulted in the identification of many genes associated with cell wall formation and organization that are up-regulated in the mature stages compared to earlier stages of development and between the small-flowered *S. australis* and the large-flowered *S. splendens* subsp. *grantii*. This shift in gene expression profiles coincides with the presence of jigsaw cells, which form a considerable proportion of the mature flowers in *Saltugilia*. None of the candidate genes known to affect cell size show differential expression between the small- and large-flowered species, but the transcriptome profiles suggest many possible candidates for controlling differences in corolla size in *Saltugilia*.

## Author contributions

Primary project design was accomplished by JL, DS and PS, while data collection was done by JL, DO, KV and RO. Data analysis was performed by JL, MG, DS and PS. All authors have approved the final version of the submitted manuscript and all agree to be accountable for all aspects of the work.

### Conflict of interest statement

The authors declare that the research was conducted in the absence of any commercial or financial relationships that could be construed as a potential conflict of interest.
