# Supplementary material for: The Phenotypic and Genetic Underpinnings of Flower Size in Polemoniaceae
Source: Front Plant Sci. 2016 Jan 5;6:1144. doi: 10.3389/fpls.2015.01144 (PMC4700140; doi:10.3389/fpls.2015.01144)
Supplement: Supplementary file 2 [file Table2.DOCX]

Supplemental Table 2. Average cell area and standard deviation for each cell type through development, including the number cells for each cell type measured.

|  | Small | | Half | | Mid | | Mature | |
| --- | --- | --- | --- | --- | --- | --- | --- | --- |
|  | Number | Mean Area | Number | Mean Area | Number | Mean Area | Number | Mean Area |
|  | *Saltugilia australis* | | | | | | | |
| Conical | 197 | 83.1 ± 23.3 | 345 | 195.6 ± 57.3 | 530 | 302.6 ± 87.6 | 335 | 454.1 ± 110.4 |
| Transition | 100 | 86.8 ± 33.6 | 128 | 204.7 ± 65.9 | 218 | 346.8 ± 155.7 | 97 | 679 ± 219.4 |
| Jigsaw | - | - | - | - | - | - | 102 | 985.9 ± 328.7 |
| Elongated | 105 | 109.5 ± 35.5 | 342 | 332.1 ± 112.4 | 300 | 678.2 ± 377.8 | 55 | 900.6 ± 580.5 |
|  | *Saltugilia caruifolia* | | | | | | | |
| Conical | 238 | 121.8 ± 50.1 | 490 | 195.4 ± 66.6 | 548 | 247.3 ± 97.7 | 457 | 430.9 ± 100.1 |
| Transition | 134 | 89.3 ± 56 | 169 | 198.1 ± 83.8 | 164 | 270.3 ± 137.5 | 221 | 547.2 ± 182.1 |
| Jigsaw | - | - | - | - | - | - | 119 | 1051.4 ± 230.4 |
| Elongated | 125 | 189.5 ± 57.8 | 367 | 445.5 ± 248.4 | 297 | 468.2 ± 281.3 | 35 | 1632.9 ± 617.9 |
|  | *Saltugilia latimeri* | | | | | | | |
| Conical | 250 | 142 ± 23.8 | 330 | 227.6 ± 55.4 | 321 | 326.8 ± 72.4 | 366 | 385.5 ± 67.7 |
| Transition | 100 | 164.9 ± 42.3 | 126 | 382.2 ± 132.1 | 113 | 406.1 ± 102.7 | 84 | 523.1 ± 1251 |
| Jigsaw | - | - | 55 | 680.6 ± 138.7 | 53 | 1227.8 ± 257.8 | 138 | 975.7 ± 294.7 |
| Elongated | 170 | 258.6 ± 68.6 | 135 | 558.5 ± 280.9 | 33 | 760.5 ± 221.9 | 69 | 1353.8 ± 560.2 |
|  | *Saltugilia splendens* subsp*. grantii* | | | | | | | |
| Conical | 340 | 132.8 ± 32.4 | 488 | 219.1 ± 49.7 | 610 | 271.2 ± 79 | 609 | 467 ± 131.5 |
| Transition | 98 | 131.4 ± 28.5 | 132 | 242.7 ± 80.3 | 185 | 267.7 ± 70.4 | 218 | 704.7 ± 222.9 |
| Jigsaw | - | - | - | - | - | - | 107 | 1493.9 ± 402.9 |
| Elongated | 430 | 185.3 ± 51 | 482 | 461.6 ± 179.9 | 507 | 533.4 ± 241.2 | 27 | 1785.5 ± 637.3 |
|  | *Saltugilia splendens* subsp*. splendens* | | | | | | | |
| Conical | 316 | 142.6 ± 38 | 456 | 212.2 ± 59.7 | 356 | 332.7 ± 117.4 | 401 | 494.9 ± 120 |
| Transition | 124 | 146. 4 ± 59.2 | 155 | 287.3 ± 127.5 | 117 | 363.6 ± 145.3 | 90 | 589.7 ± 140.1 |
| Jigsaw | - | - | - | - | - | - | 61 | 1352.9 ± 384.7 |
| Elongated | 251 | 385.2 ± 186.6 | 247 | 578 ± 329.1 | 148 | 861.7 ± 542.3 | - | - |

|  | Small | | Half | | Mid | | Mature | |
| --- | --- | --- | --- | --- | --- | --- | --- | --- |
|  | Number | Mean Area | Number | Mean Area | Number | Mean Area | Number | Mean Area |
|  | *Saltugilia splendens* subsp*. splendens* (field) | | | | | | | |
| Conical | 328 | 130.5 ± 30.8 | 399 | 241.2 ± 61.8 | 580 | 315 ± 74.1 | 537 | 423.8 ± 99.5 |
| Transition | 105 | 180.4 ± 42.8 | 150 | 284.6 ± 89.4 | 166 | 426.3 ± 136.3 | 133 | 696.5 ± 159.5 |
| Jigsaw | - | - | 156 | 701.9 ± 376.7 | 180 | 1048 ± 430.1 | 162 | 1446.7 ± 429.8 |
| Elongated | 381 | 270.5 ± 81.1 | 224 | 651.1 ± 385.1 | 98 | 1670.9 ± 538.5 | 83 | 1716.7 ± 609.9 |
|  | *Gilia stellata* | | | | | | | |
| Conical | 254 | 118.2 ± 49.6 | 213 | 169.8 ± 63 | - | - | 273 | 295.4 ± 82 |
| Transition | 161 | 178.2 ± 106.4 | 133 | 288.9 ± 137.3 | - | - | 131 | 479.3 ± 169.6 |
| Jigsaw | - | - | - | - | - | - | 112 | 1274.8 ± 369.8 |
| Elongated | 214 | 315.1 ± 153.2 | 198 | 658 ± 489.8 | - | - | 32 | 2139.2 ± 610.7 |
|  | *Gilia brecciarum* subsp*. brecciarum* | | | | | | | |
| Conical | 90 | 99.7 ± 23.6 | 40 | 234.5 ± 41.8 | - | - | 79 | 242.4 ± 53.3 |
| Transition | 23 | 186.6 ± 70.2 | 18 | 387.2 ± 143.5 | - | - | 15 | 601.5 ± 115.6 |
| Jigsaw | - | - | - | - | - | - | 3 | 1916.1 ± 406.4 |
| Elongated | - | - | 1 | 1554.6 | - | - | 1 | 2863.3 |
