# Supplementary material for: The Phenotypic and Genetic Underpinnings of Flower Size in Polemoniaceae
Source: Front Plant Sci. 2016 Jan 5;6:1144. doi: 10.3389/fpls.2015.01144 (PMC4700140; doi:10.3389/fpls.2015.01144)
Supplement: Supplementary file 4 [file Table4.docx]

Table 4. Cell number estimates for each cell type and overall flower by taking the mean bounding box of each measured

cell. Total physical length of each cell type was then divided by the mean to obtain estimates of the total number of cells necessary to

contribute to overall length of each cell type. Total cell counts denoted with * had missing data for at least one cell type.

| Species/Plant | Plant # | Size | Conical | Transition | Jigsaw | Elongated | Total |
| --- | --- | --- | --- | --- | --- | --- | --- |
| *Gilia brecciarum* subsp. *brecciarum* | 2 | Small | 107 | 0 | 0 | 0 | 107* |
| *Gilia brecciarum* subsp. *brecciarum* | 2 | Half | 149 | 34 | 0 | 36 | 219 |
| *Gilia brecciarum* subsp. *brecciarum* | 2 | Full | 103 | 54 | 9 | 16 | 181 |
| *Gilia stellata* | 1 | Full | 124 | 11 | 40 | 13 | 187 |
| *Gilia stellata* | 2 | Small | 144 | 19 | 0 | 18 | 181 |
| *Gilia stellata* | 2 | Full | 145 | 16 | 35 | 18 | 213 |
| *Gilia stellata* | 3 | Small | 90 | 14 | 0 | 48 | 152 |
| *Gilia stellata* | 3 | Half | 125 | 20 | 32 | 9 | 186 |
| *Gilia stellata* | 3 | Full | 127 | 12 | 35 | 4 | 177 |
| *Gilia stellata* | 4 | Small | 87 | 13 | 0 | 36 | 137 |
| *Gilia stellata* | 4 | Half | 128 | 4 | 0 | 46 | 177 |
| *Gilia stellata* | 4 | Full | 124 | 8 | 28 | 29 | 189 |
| *Saltugilia australis* | 1 | Small | 131 | 35 | 0 | 16 | 182 |
| *Saltugilia australis* | 1 | Half | 178 | 24 | 0 | 37 | 239 |
| *Saltugilia australis* | 1 | Mid | 147 | 16 | 0 | 47 | 210 |
| *Saltugilia australis* | 2 | Mid | 177 | 14 | 0 | 50 | 241 |
| *Saltugilia australis* | 2 | Full | 142 | 17 | 84 | 1 | 244 |
| *Saltugilia australis* | 3 | Small | 155 | 0 | 0 | 0 | 155* |
| *Saltugilia australis* | 3 | Half | 186 | 12 | 0 | 46 | 244 |
| *Saltugilia australis* | 3 | Mid | 179 | 14 | 0 | 52 | 245 |
| *Saltugilia australis* | 3 | Full | 162 | 13 | 31 | 36 | 242 |
| *Saltugilia australis* | 4 | Small | 114 | 38 | 0 | 82 | 233 |
| *Saltugilia australis* | 4 | Half | 123 | 12 | 0 | 48 | 183 |
| *Saltugilia australis* | 4 | Mid | 136 | 15 | 0 | 54 | 204 |
| *Saltugilia australis* | 4 | Full | 133 | 15 | 19 | 43 | 210 |
| *Saltugilia caruifolia* | 1 | Small | 223 | 8 | 0 | 24 | 256 |
| *Saltugilia caruifolia* | 1 | Half | 237 | 85 | 0 | 20 | 342 |
| *Saltugilia caruifolia* | 1 | Mid | 215 | 26 | 0 | 49 | 289 |
| *Saltugilia caruifolia* | 1 | Full | 193 | 26 | 30 | 29 | 278 |
| *Saltugilia caruifolia* | 2 | Small | 136 | 127 | 0 | 36 | 300 |
| *Saltugilia caruifolia* | 2 | Half | 212 | 5 | 0 | 42 | 258 |
| *Saltugilia caruifolia* | 2 | Mid | 214 | 25 | 0 | 66 | 305 |
| *Saltugilia caruifolia* | 2 | Full | 161 | 7 | 58 | 23 | 249 |
| *Saltugilia caruifolia* | 3 | Half | 211 | 31 | 0 | 26 | 268 |
| *Saltugilia caruifolia* | 3 | Mid | 246 | 21 | 0 | 78 | 344 |
| *Saltugilia caruifolia* | 3 | Full | 196 | 6 | 66 | 6 | 275 |
| *Saltugilia caruifolia* | 4 | Small | 179 | 63 | 0 | 5 | 247 |
| *Saltugilia caruifolia* | 4 | Half | 227 | 10 | 0 | 49 | 286 |
| *Saltugilia caruifolia* | 4 | Mid | 251 | 2 | 0 | 54 | 308 |
| *Saltugilia caruifolia* | 4 | Full | 176 | 0* | 77 | 21 | 275 |
| *Saltugilia latimeri* | 1 | Half | 101 | 20 | 14 | 16 | 150 |
| *Saltugilia latimeri* | 1 | Mid | 116 | 16 | 15 | 0* | 147 |
| *Saltugilia latimeri* | 1 | Full | 110 | 22 | 26 | 17 | 175 |
| *Saltugilia latimeri* | 2 | Small | 105 | 9 | 0 | 34 | 148 |
| *Saltugilia latimeri* | 2 | Half | 128 | 11 | 11 | 29 | 179 |
| *Saltugilia latimeri* | 2 | Mid | 93 | 3 | 0 | 87 | 184 |
| *Saltugilia latimeri* | 2 | Full | 116 | 38 | 17 | 26 | 197 |
| *Saltugilia latimeri* | 3 | Small | 105 | 36 | 0 | 22 | 163 |
| *Saltugilia latimeri* | 3 | Half | 119 | 9 | 0 | 42 | 170 |
| *Saltugilia latimeri* | 3 | Full | 152 | 28 | 32 | 20 | 233 |
| *Saltugilia latimeri* | 4 | Mid | 120 | 21 | 18 | 0 | 159 |
| *Saltugilia latimeri* | 4 | Full | 165 | 0* | 45 | 26 | 236 |
| *Saltugilia splendens* subsp. *grantii* | 1 | Small | 336 | 0* | 0 | 86 | 422 |
| *Saltugilia splendens* subsp. *grantii* | 1 | Half | 274 | 21 | 0 | 84 | 379 |
| *Saltugilia splendens* subsp. *grantii* | 1 | Mid | 254 | 13 | 0 | 137 | 404 |
| *Saltugilia splendens* subsp. *grantii* | 1 | Full | 344 | 97 | 112 | 13 | 567 |
| *Saltugilia splendens* subsp. *grantii* | 2 | Mid | 192 | 14 | 0 | 154 | 360 |
| *Saltugilia splendens* subsp. *grantii* | 2 | Full | 225 | 77 | 91 | 31 | 424 |
| *Saltugilia splendens* subsp. *grantii* | 3 | Small | 214 | 10 | 0 | 134 | 358 |
| *Saltugilia splendens* subsp. *grantii* | 3 | Half | 257 | 6 | 0 | 140 | 403 |
| *Saltugilia splendens* subsp. *grantii* | 3 | Mid | 245 | 4 | 0 | 165 | 413 |
| *Saltugilia splendens* subsp. *grantii* | 3 | Full | 272 | 63 | 47 | 54 | 436 |
| *Saltugilia splendens* subsp. *grantii* | 4 | Small | 183 | 6 | 0 | 128 | 317 |
| *Saltugilia splendens* subsp. *grantii* | 4 | Half | 168 | 18 | 0 | 132 | 318 |
| *Saltugilia splendens* subsp. *grantii* | 4 | Mid | 261 | 23 | 0 | 198 | 482 |
| *Saltugilia splendens* subsp. *grantii* | 4 | Full | 235 | 27 | 76 | 77 | 415 |
| *Saltugilia splendens* subsp. *splendens* | 1 | Small | 153 | 14 | 0 | 28 | 196 |
| *Saltugilia splendens* subsp. *splendens* | 1 | Half | 176 | 13 | 0 | 63 | 252 |
| *Saltugilia splendens* subsp. *splendens* | 1 | Full | 122 | 27 | 75 | 0 | 224 |
| *Saltugilia splendens* subsp. *splendens* | 2 | Half | 166 | 10 | 0 | 48 | 224 |
| *Saltugilia splendens* subsp. *splendens* | 3 | Small | 179 | 58 | 0 | 6 | 243 |
| *Saltugilia splendens* subsp. *splendens* | 3 | Half | 110 | 36 | 0 | 11 | 157 |
| *Saltugilia splendens* subsp. *splendens* | 3 | Mid | 155 | 20 | 0 | 50 | 224 |
| *Saltugilia splendens* subsp. *splendens* | 3 | Full | 155 | 9 | 53 | 0 | 217 |
| *Saltugilia splendens* subsp. *splendens* | 4 | Small | 150 | 17 | 0 | 39 | 206 |
| *Saltugilia splendens* subsp. *splendens* | 4 | Half | 124 | 12 | 0 | 53 | 189 |
| *Saltugilia splendens* subsp. *splendens* | 4 | Mid | 137 | 14 | 0 | 58 | 209 |
| *Saltugilia splendens* subsp. *splendens* | 4 | Full | 136 | 11 | 67 | 8* | 222 |
| *Saltugilia splendens* subsp. *splendens* (field) | 1 | Small | 180 | 12 | 0 | 50 | 242 |
| *Saltugilia splendens* subsp. *splendens* (field) | 1 | Mid | 201 | 19 | 51 | 16 | 287 |
| *Saltugilia splendens* subsp. *splendens* (field) | 1 | Full | 216 | 14 | 64 | 31 | 325 |
| *Saltugilia splendens* subsp. *splendens* (field) | 2 | Small | 177 | 15 | 0 | 51 | 243 |
| *Saltugilia splendens* subsp. *splendens* (field) | 2 | Half | 165 | 20 | 0 | 68 | 253 |
| *Saltugilia splendens* subsp. *splendens* (field) | 2 | Mid | 177 | 25 | 45 | 25 | 272 |
| *Saltugilia splendens* subsp. *splendens* (field) | 2 | Full | 197 | 26 | 58 | 0 | 281 |
| *Saltugilia splendens* subsp. *splendens* (field) | 3 | Small | 178 | 17 | 0 | 55 | 250 |
| *Saltugilia splendens* subsp. *splendens* (field) | 3 | Half | 220 | 17 | 37 | 40 | 313 |
| *Saltugilia splendens* subsp. *splendens* (field) | 3 | Mid | 229 | 17 | 57 | 8 | 311 |
| *Saltugilia splendens* subsp. *splendens* (field) | 3 | Full | 244 | 16 | 58 | 32 | 350 |
| *Saltugilia splendens* subsp. *splendens* (field) | 4 | Small | 196 | 0* | 0 | 49 | 244 |
| *Saltugilia splendens* subsp. *splendens* (field) | 4 | Half | 189 | 15 | 39 | 30 | 273 |
| *Saltugilia splendens* subsp. *splendens* (field) | 4 | Mid | 231 | 11 | 41 | 30 | 313 |
| *Saltugilia splendens* subsp. *splendens* (field) | 4 | Full | 188 | 13 | 63 | 24 | 288 |
