# Supplementary figures and images for: The Phenotypic and Genetic Underpinnings of Flower Size in Polemoniaceae
Source: Front Plant Sci. 2016 Jan 5;6:1144. doi: 10.3389/fpls.2015.01144 (PMC4700140; doi:10.3389/fpls.2015.01144)

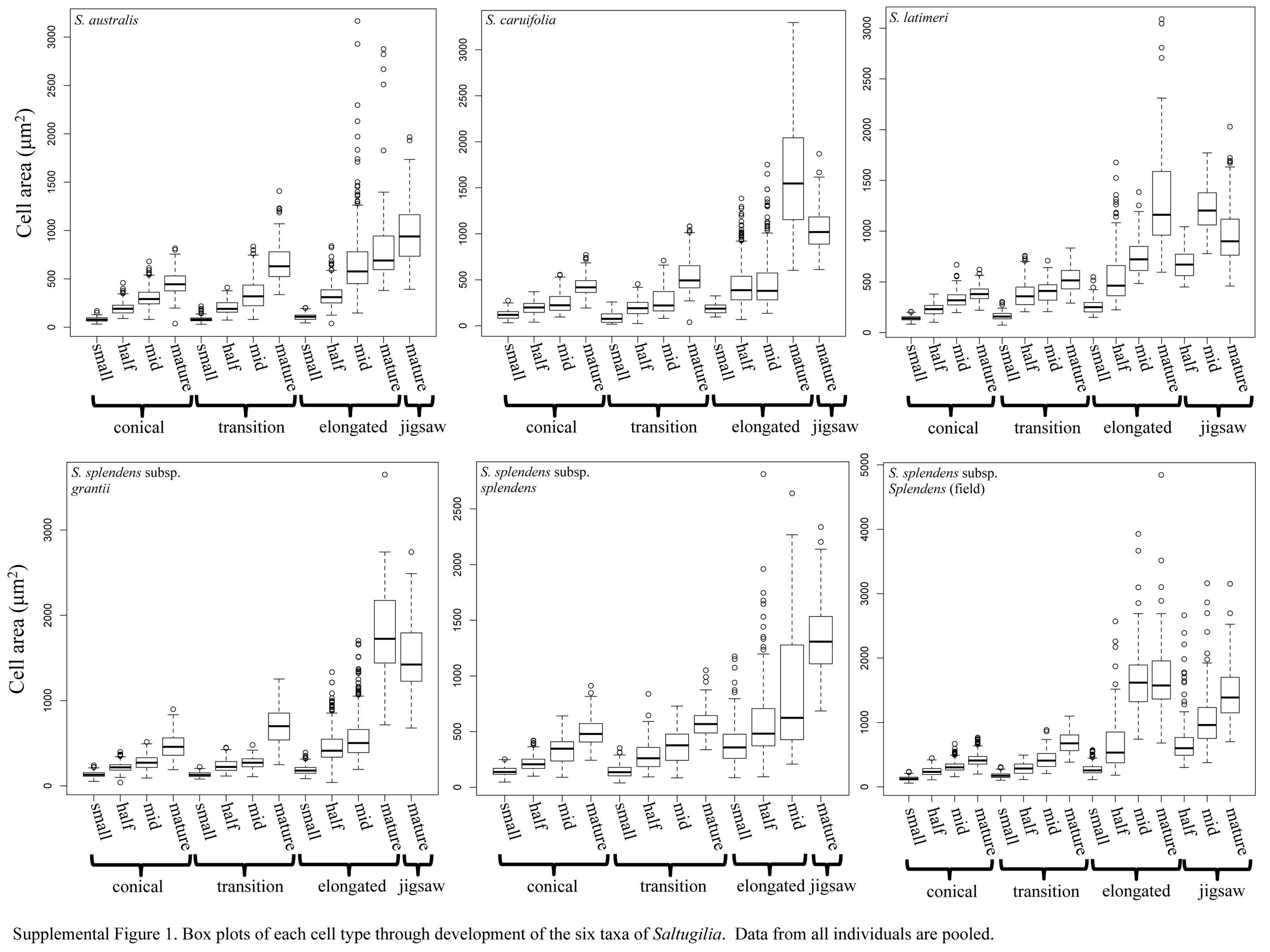

Supplement: Supplementary file 8 [file Image1.TIF]
